# Force output in giant-slalom skiing: A practical model of force application effectiveness

**DOI:** 10.1371/journal.pone.0244698

**Published:** 2021-01-14

**Authors:** Matt R. Cross, Clément Delhaye, Jean-Benoit Morin, Maximilien Bowen, Nicolas Coulmy, Frédérique Hintzy, Pierre Samozino

**Affiliations:** 1 Laboratoire Interuniversitaire de Biologie de la Motricité, Université Savoie Mont Blanc, Chambéry, France; 2 Département Scientifique et Sportif, Fédération Française de Ski, Annecy, France; 3 Inter-University Laboratory of Human Movement Biology, Univ Lyon, UJM-Saint-Etienne, Saint-Étienne, France; University of Innsbruck, AUSTRIA

## Abstract

Alpine ski racers require diverse physical capabilities. While enhanced force production is considered key to high-level skiing, its relevance is convoluted. The aims of this study were to i) clarify the association between performance path length and velocity, ii) test the importance of radial force, and iii) explore the contribution of force magnitude and orientation to turn performance. Ski athletes (*N* = 15) were equipped with ski-mounted force plates and a global navigation satellite system to compute the following variables over 14 turns: path length (*L*), velocity normalized energy dissipation [Δ*e*_mech_/*v*_in_], radial force [*F*_r_], total force (both limbs [*F*_tot_], the outside limb, and the difference between limbs), and a ratio of force application (*RF* = *F*_r_/*F*_tot_). Data were course-averaged or separated into sectional turn groupings, averaged, and entered into stepped correlation and regression models. Our results support Δ*e*_mech_/*v*_in_ as a discriminative performance factor (*R*^2^ = 0.50–0.74, *p <* .003), except in flat sections. Lower course times and better Δ*e*_mech_/*v*_in_ were associated with greater *F*_r_ (*R*^2^ = 0.34–0.69 and 0.31–0.52, respectively, *p <* .032), which was related to both *F*_tot_ and *RF* (*β* = 0.92–1.00 and 0.63–0.81, respectively, *p <* .001) which varied in predictive order throughout the sections. *F*_tot_ was associated with increased outside limb force and a more balanced contribution of each limb (*β* = 1.04–1.18 and -0.65– -0.92, respectively, *p <* .001). *F*_r_ can be improved by either increasing total force output or by increasing technical effectiveness (i.e., proportionally more force radially) which should increase the trajectories available to the skier on the ski course.

## 1 Introduction

In alpine ski racing athletes must negotiate a demanding course under variable constraints including event specific gate setups, challenging terrain, snow and weather conditions. Podium places are often separated by mere fractions of a second, which highlights the importance of chasing small improvements in performance. Better understanding of the contributors to performance in this complex sport should provide a basis to better prepare athletes, and consequently produce worthwhile improvements in technique and conditioning.

Determining which capabilities separate high-level skiers from their lower-level counterparts is challenging. Skiing motion is essentially a result of utilizing the potential energy possessed at the start of the course, by converting this energy into either kinetic energy or dissipating it (via ski−snow and aerodynamic friction) within the trajectory limits imposed by gates. An athlete who possesses the physical and technical capabilities necessary to effectively manipulate and, ultimately, minimize energy loss within the marked course is at an advantage [1–3]. In its most basic form, to turn and successfully negotiate a course, athletes must apply force onto the snow via their ski-specific equipment. While the contribution of other external forces might play a role in some disciplines (e.g., minimizing aerodynamic drag is a dominant factor in speed events [4]), the main changeable mechanism of skiing is the production of force onto the snow, which induces snow-reaction forces (*SRF*) associated to changes in skier motion or as a product of changes in the ski-snow interaction (i.e., changes in energy dissipation). The interplay of force-output with skiing performance is complex, and while it is impossible to ski effectively without a certain capability to generate and appropriately apply force to the snow, force applied at a given moment can serve to improve or worsen the instantaneous performance of a skier [5]. For these reasons, force output in skiers has attracted interest in applied research [4] and conditioning practice [6].

While our understanding of skiing biomechanics has advanced considerably in recent years [7], the physical, technical, and tactical determinants of skiing performance remain largely unclear. Course performance (i.e., time) can be contextualized as comprising multiple distinct but interrelated sections, in which there are intertwining approaches to minimizing time: i) selecting an advantageous and more direct route between gates, or ii) preferencing a longer trajectory that enables greater speeds to be attained or maintained (depending on the characteristics upon entering the section) [5]. While the former approach is intuitive, since the distance traveled by the athlete is reduced, the ensuing tighter turns might dissipate more energy [2]. The latter approach is typically associated with a longer path between gates [2, 8], but this detractor can be overcome if enough velocity is gained within the section or maintained (i.e., less energy dissipated) [1–3, 9]. Moreover, a greater sectional velocity directly influences performance in coming turns, which can be an advantage not clearly apparent within the turn itself (provided the additional velocity can be managed effectively). In slalom and giant-slalom (GS) there is some evidence to show that strategies enabling an elevated turn velocity appear preferable to a more direct route between gates [10]. Summarizing the recent stance on the approach, Spörri, Kröll [11] suggest beginning turns earlier [3, 9], and smoothly applying force [9] and ‘carving’ the skis (i.e., rather than skidding) [1–3, 9] as strategies to elevate velocity and performance in technical skiing disciplines. Unfortunately, our understanding of this phenomenon is drawn from a small pool of studies featuring specific and constrained external conditions. As such, it is possible that different results might be observed across disciplines, and with different course characteristics [12, 13]. In any case, attaining and maintaining high sectional velocities is limited by course constraints [14], technique [15], tactical approaches [16], and the physical competency of the athlete.

According to Newton’s laws of motion, turns performed at higher velocities and/or with smaller turn radii (*r*) require elevated radial force output compared to slower and straighter turns (i.e., radial force [*F*_r_] = velocity^2^/*r*). On the one hand, the same *F*_r_ can theoretically be observed in various situations per the tactical approach to the turn mediated by the characteristics at entry (notably, entry velocity [2]). For example, two sections might display inversely proportionate velocity and turn radii (i.e., higher velocity and lower radius, and the inverse) and display similar force, and dissipation characteristics. On the other hand, athletes possessing an elevated capability to generate *F*_r_ might be able to turn tighter at a given velocity or turn at a higher velocity with a given turn radius without needing to dissipate unnecessary additional energy. This ability to maximize *F*_r_ is well represented in the ‘carving’ technique, where anecdotally a large proportion of force being produced is directly turning the athlete along a curved trajectory (i.e., acting perpendicular to the tangential direction of the ski trajectory; *F*_r_). In carving, a large proportion of the turning phase is performed with the skis on edge and penetrating the snow surface, without a substantial lateral skidding component and associated minimal dissipation of energy [17]. Reid, Haugen [18] describe the phenomenon well, where carving is characterized by a reduced ‘attack angle’ between the longitudinal axis of the ski, and the velocity vector of its center. Practically, increasing the attack angle results in skidding, with the inverse presenting the appearance of all points of the ski edge ‘carving’ through the same point on the snow. The interplay between carving and skidding is complicated, with some proportion of the turn inevitably being performed in conditions of skidding, and being mediated by a host of other factors such as the interaction between the environment and the ski geometry [19]. Albeit a simplification, carving represents the most obvious way of attaining a greater velocity without unnecessary dissipation of energy [1–3, 9], provided the conditions permit it. However, athletes might deviate from carving in certain situations as a tactical choice (e.g., skidding to dissipate energy and avoid mistakes), or as a product of adopting a more direct trajectory, spending longer in the fall-line, and turning with a smaller radius [19]. Nevertheless, without a capability to produce and control high levels of *F*_r_ athletes might reduce their strategic options, with the result being, on average, a lesser performance than otherwise possible from choosing strategies to disperse energy or a less optimal line.

While in numerous sport movements (e.g., sprint running, cycling, or jumping) the maximal velocity is related to the highest kinetic work the athlete can produce, skiing velocity is a result of efficiently managing the transformation of energy [16]. Consequently, maximal velocity corresponds to the highest value at which the athlete can ski while respecting the trajectory limits imposed by gates and without losing control and risking injury; this phenomenon is widely known as the ‘velocity barrier’ [2]. While not widely researched, this barrier is likely the result of intertwining unmodifiable factors (e.g., course setting and snow quality), and physiological and psychological systems (e.g., perceptual, trajectory targeting). Since it appears that the main mechanism behind skiing is the manipulation of *SRF*, conceivably athletes with enhanced force output (notably *F*_r_) might possess, *ceteris paribus*, a raised ‘barrier’ compared to their counterparts and, consequently, elevated control at higher velocities, and a model terminus of better course performance. In other words, increasing athlete force output capability is one theoretical means of improving performance during turns made at the limits of athletic ability. Assuming generating elevated *F*_r_ is important to ski-racing performance, the two main approaches to improving this variable would be increasing the total magnitude of force applied to the snow or applying a greater proportion of total capacity in the radial direction. In any case, at present the differentiation between total force output and that applied radially is unclear.

There is no consensus on the predictive nature of various force-output characteristics, other than the timing and event specific application of force being of higher importance than non-descript output [5]. For example, Supej, Kipp [2] showed that slalom skiing *SRF* magnitudes did not clearly differentiate between athletes but rather acutely high values during turns were associated with elevated energy dissipation across the cohort. In a small homogenous sample, Reid [19] reported a strong association between turn time and *SRF* during a single 10 m slalom turn, but not when gate distance was increased to 13 m. Furthermore, athletes who adopt a ‘carving’ technique, as opposed to a more traditional technique with shorter trajectories, ski faster but exhibit non-dissimilar average *SRF* [15, 20]. One interpretation is that while an enhanced capacity for total force output may be advantageous, what is more important is the way in which force is applied to the snow; notably, a greater proportion of force produced to carve the ski (i.e., elevated *F*_r_) instead of total output in less advantageous techniques (i.e., elevated energy loss). There is relatively little information regarding the technical and physical parameters underlying *SRF* output in skiing, and a wide variety of measurement techniques (direct vs. indirect force measurement) and methodological approaches (high-level athletes performing race-pace trials [21] vs. recreational athletes performing free-ski turns [20]) render synthesizing findings challenging. Moreover, a common acknowledged limitation is the extrapolation of a minor representation of the course (e.g., 1–2 turns) to overall performance [3]. Sectional times in ski racing are notoriously variable (e.g., ~10% in high-level athletes) [22] and competition courses are comprised of external constraints that can vary substantially turn-by-turn (e.g., high- and low-inclination sections), which highlights the value in drawing conclusions from a wider breadth of the course.

In alpine skiing, *SRF* can be estimated via pressure insoles [23] or specialized force platforms [20], and using inverse dynamics via video [2] or Global Navigation Satellite System (GNSS) [4] in combination with other micro-technologies [11]. Force platforms remain the gold-standard method, but difficulty integrating such technology into skiing practice has substantially limited their implementation in research. Furthermore, until recently technology lacked optimal data capture capabilities (e.g., low sample frequencies) and typically featured somewhat invasive designs (e.g., increased clip-in height, weight, and modifications to the rigidity of the ski) that inevitably degraded measurement validity. Moreover, while biomechanical analysis in alpine skiing appears in the published literature in the 1980s [5], modifications to the ruling structures, slope preparation, and ski-binding technology mean that the biomechanical environment in which the athletes perform has changed markedly [24]. Consequently, despite a recent thrust in ski-research efforts coinciding with the development of portable technologies (e.g., high frequency, portable, low-cost inertial units), there is still a paucity of published information on the topic [16]. Ski-force-platform systems have benefited from similar technological advances [25], but an inability to determine the snow referential complicates separating the proportion of force output applied effectively during a turn (i.e., *F*_r_). While *F*_r_ can be computationally separated from total force when measured from an inverse dynamics model applied to center of mass (COM) kinematics, the total *SRF* might not exactly track that produced by the limbs in isolation. Consequently, a combination of approaches might represent the best means of providing a holistic view of *SRF* output characteristics in skiing.

The aims of the present study were to i) clarify the association between skiing performance and optimizing trajectories and/or maximizing velocity, ii) test the importance of *F*_r_ in skiing performance, and iii) further explore the importance of force magnitude and/or orientation of force-output in skiing turn performance. To this end, we measured force output and positional data using a combination of recently validated ski-specialized force plates [25] and a sports GNSS system on high-level skiers while they performed a race-paced short GS course. Based on previous research, our hypothesis was that performance would be well predicted by a capability to minimize energy dissipation, associated with elevated *F*_r_ output. We further predicted that *F*_r_ would be largely determined by both the capability to produce force and orient it radially. Lastly, provided the total force produced by the lower limbs is a limiting factor for *F*_r_, we assumed that athletes who presented greater total force would do so by producing more force to both the outside and inside limbs.

## 2 Materials and methods

### 2.1 Participants and protocol

Alpine skiers with diverse backgrounds (*N* = 16), training histories, and performance levels (i.e., ski monitors and club participants to current World Cup competitors) participated in this study (age 26±5 yr; stature 1.79±0.06 m; body mass [BM] 84.0±8.1 kg; course time [*T*] 29.14±1.60 s) over a 5-week period in winter. Athletes were devoid of any injuries that would affect their ability to participate fully in the testing. Before testing commenced, athletes were instructed on the testing procedures, allowed an opportunity to answer questions, and then provided written consent regarding their participation in the study. All operational procedures were completed within the ethical requirements of the Fédération Française de Ski and the Code of Ethics of the World Medical Association (Declaration of Helsinki), and approved by the ethics committee of the University Savoie Mont Blanc.

**Fig 1:**
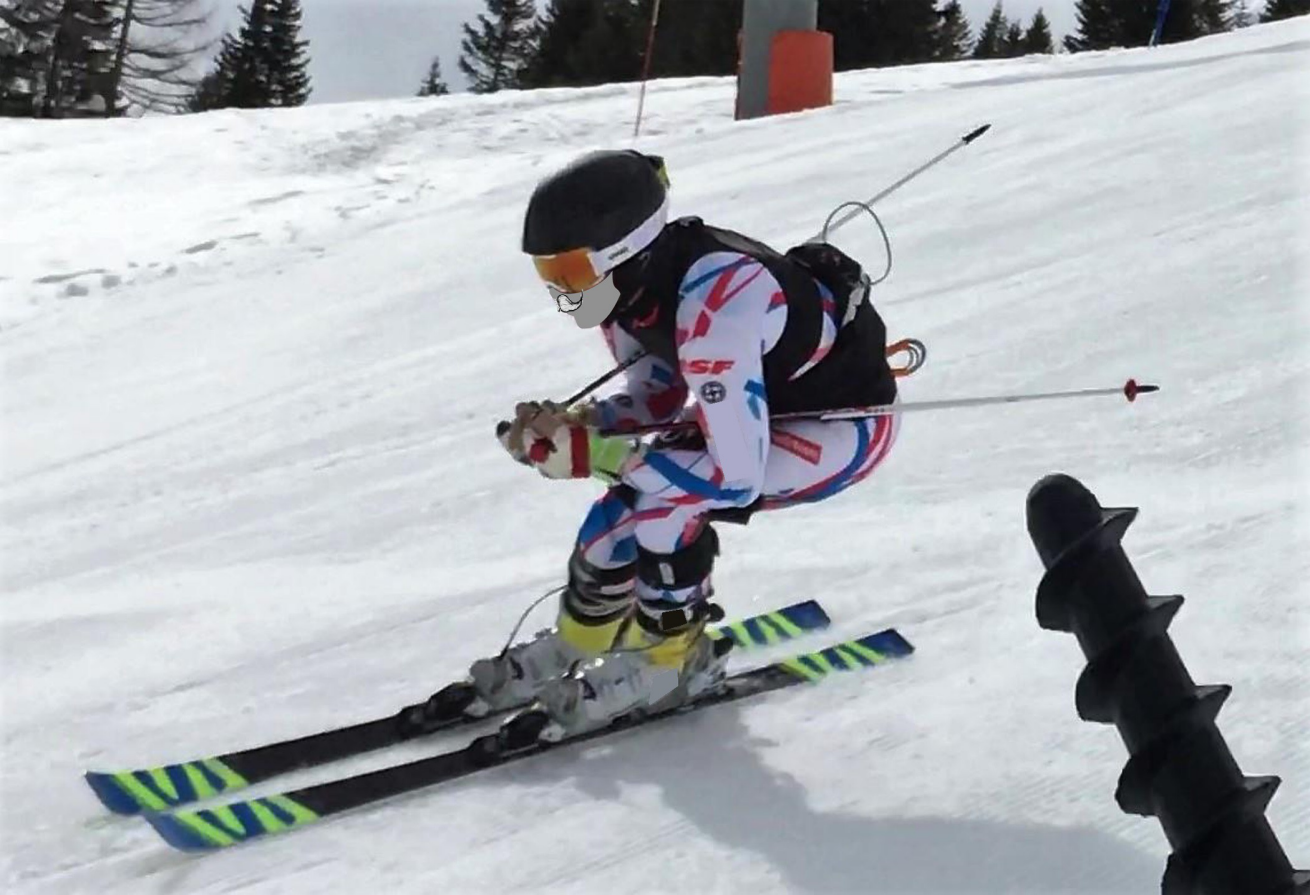
Skier equipped with experimental technology. Pictured is a skier performing a trial, while equipped with boot-mounted force platforms, wired to an acquisition card carried in a small bag around the waist with a GNSS unit secured inside.

Athletes were equipped with a GNSS system and with specialized force platforms attached to race-boots and skis (see Fig 1), and then performed one warm-up of free skiing starting from the top of the mountain through the experimental course. Athletes then performed a competition-style personalized warm-up, followed by 2–3 familiarization runs. Once comfortable and familiar with the course and equipment, athletes proceeded to a small hut near the starting gate and performed a short calibration procedure for the equipment on solid ground (detailed in *Section 3*.*3*.*1*.).

Athletes performed skiing trials on an 18-gate GS course, interspersed with approximately 15 minutes of passive rest (including non-fatiguing calibration procedures). The number of trials was decided based on whether the athlete reported submaximal effort or undue mistakes during their previous trial, corroborated by the experimenters, up to a maximum of 3 trials. During their trials, athletes took up place at the top of the course, the starting gate was set for timing, and athletes began their trial in their own time. The athletes were instructed to ski at maximum pace and were questioned on their efforts post-trial. Where more than one trial was performed, the trial with the fastest *T* was selected for final analysis.

### 2.2 Equipment and course characteristics

#### 2.2.1 Course setting and monitoring environmental conditions

**Fig 2:**
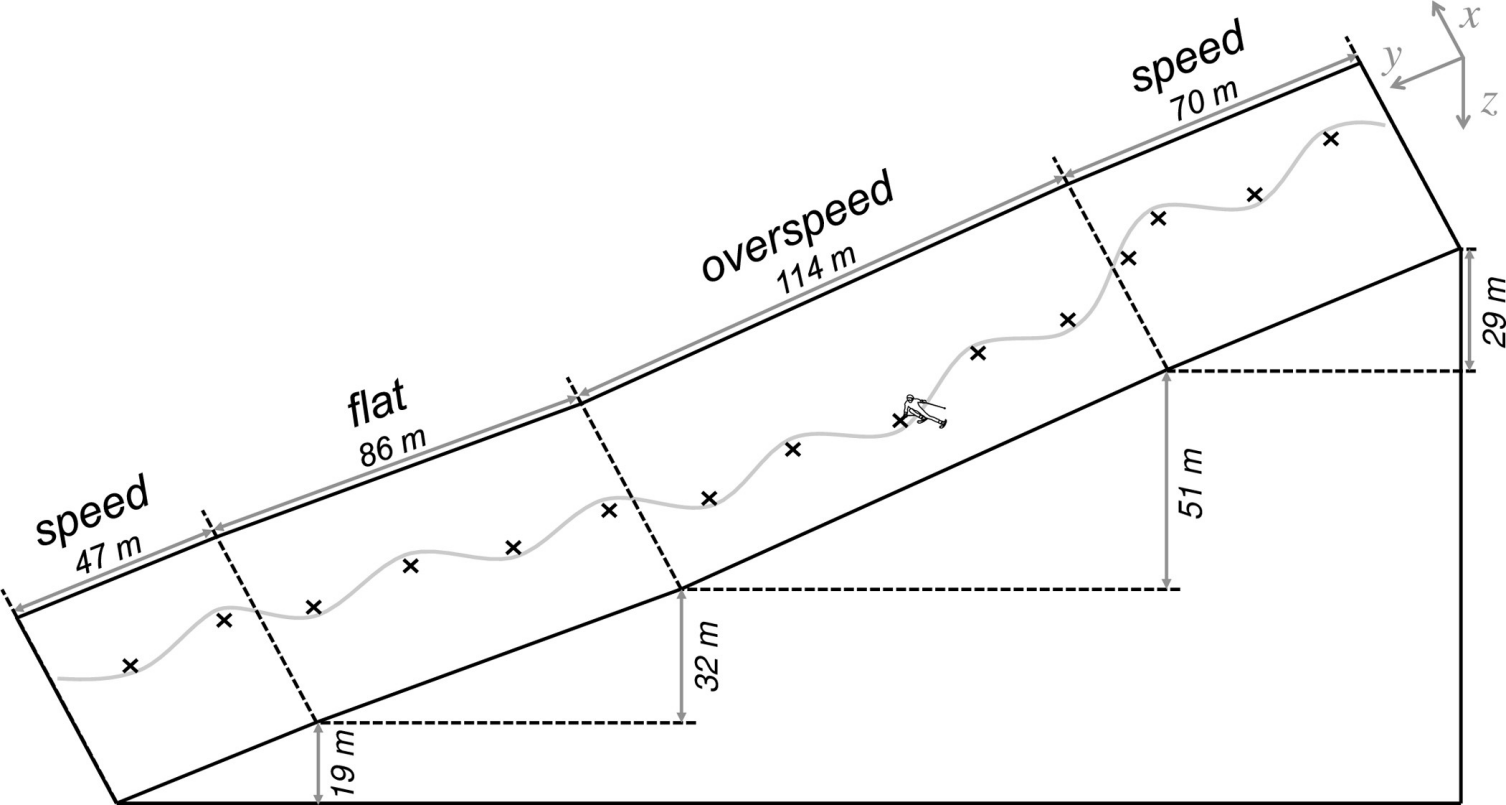
Course representation. Depicted is a skier (not to scale, for clarity) traversing the course setup. ×, gates; grey line, athlete trajectory through analyzed gates (expanded for clarity); dashed lines represent the separation of the various analysis sections (speed, overspeed, and flat), with the approximate section characteristics (i.e., latitude and altitude) displayed in the corresponding axes (y- and z-axis, respectively). Note: since no course mapping was performed, section characteristics represent the average value between the turn-switches demarcating the beginning and end of each section, across all athletes and trials.

Throughout the duration of experimentation, snow quality (hardness, crystalline structure, and temperature) was monitored, as well as environmental conditions (temperature, wind, and visibility). The average temperature was ~1°C, with the nights preceding testing having temperatures low enough to freeze snow on the course. The course setting was initially performed by a high-level ski federation coach, adhering to a GS setup: 18 gates, ~25° slope relief (see Fig 2). Netting poles were placed along the periphery of the slope which remained in place throughout the duration of the study, and to corroborate gate positions they were measured with respect to at least two external poles and the gates preceding them. Where necessary, measurements were made to additional immoveable points (pylons and ski-track mountings). As such, for each point we were able to triangulate the initial position, and corroborated using both external and internal points, and accurately replicate the course setting throughout experimentation.

#### 2.2.2 Ski equipment

For each trial, skiers were equipped with the same pair of race skis (model: LAB X-RACE GS, Salomon, Annecy, France; radius: 30 m; length: 1.93 m) and bindings (model: X19 LAB, Salomon, Annecy, France), the bases and edges of which were prepared by a ski technician before each experimental day. The skis were fitted with a binding plate fabricated to accommodate the elongated clip-in length resulting from the sensor attachment (+60 mm). Athletes were provided with a selection of race model ski boots (model: X LAB+ 140 WC, Salomon, Annecy, France), in 1 size (10 mm) increments between sizes 24.5 and 28.5. Where preferred, use of personal boot-liners (and/or tongues) was allowed. Athletes used their own race suits (or were supplied with the appropriately sized suit) and other International Ski Federation (FIS) approved race clothing and protective gear (e.g., helmet, goggles, and back protectors).

#### 2.2.3 Positional measurement

Athletes were equipped with a portable (~20 g) sports GNSS system (model: Stadium tracker, Mac-Lloyd Sport, Paris, France), which was mounted in a small hip bag attached at approximately the height of COM (L5 vertebrae). The GNSS collected positional data from both Russian (GLONASS) and American (GPS) satellite constellation systems and featured a 9-axis accelerometer and IMU system (18 and 100 Hz, respectively; end data down sampled to 10 Hz). Data were collected using the manufacturer supplied software which featured a fusion algorithm designed to improve positioning accuracy by combining satellite derived data with that from the inertial sensors [26, 27].

Time between the start and end of the run was measured using an FIS approved wireless system (model: ‘Basic wireless solution’, Tag Heuer, La Chaux-de-Fonds, Switzerland), comprising of a starting-gate trigger and a dual-beam photo-voltaic cell set at approximately thigh height at the final gate in the course. For redundancy and data corroboration, two cameras (model: Hero 5, GoPro, San Mateo, USA) were used to record each skier: one operated by a researcher filming gates 6 through 9, and the second held by another researcher who followed the skier at approximately 5–10 m. Both cameras were affixed to handheld stabilizers (model: G5, FeiyuTech, Guilin, China). Cameras were manually time synchronized with each trial by filming part of the calibration process.

*2*.*2*.*3*.*1*. *GNSS validity and reliability*. GNSS units are widely used in the sporting literature [26], and while they can provide worthwhile data regarding a variety of relevant metrics for skiing performance in a practical and easy manner [28], accuracy is system dependent and highly variable [29]. To test the accuracy of our system and methodological approach, we performed two separate analyses: i) a quasi-validity study, in which we compared key variables from the system against a criterion Real-Time Kinematic (RTK) system (see S1 Material), and ii) a test of intra-athlete reliability, where key kinematic variables were compared in athletes who completed two maximum effort trials of the current testing protocol (*N* = 6). Lastly, as a strategy to reduce the potential error associated with our units, we selected an analysis approach that examined only turn-averaged data that was subsequently further averaged over a minimum of three turns [30].

#### 2.2.4 Force measurement

**Fig 3:**
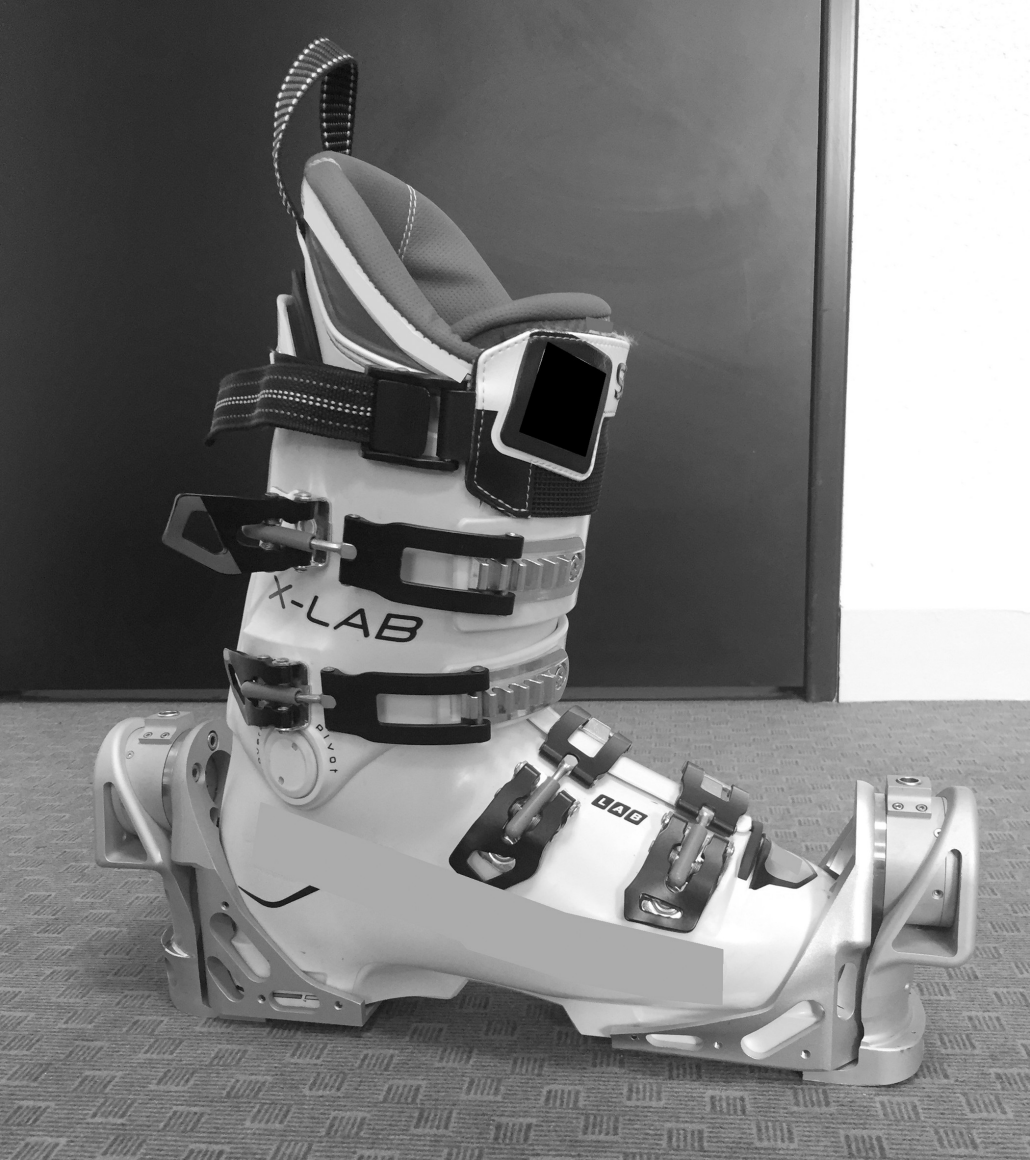
Ski-specialized force plates. Pictured are the specialized force-plate devices used to collect the primary SRF data in this study, attached to a pair of standardized boots used during the experiment. The novelty of these devices in contrast to previous designs is that the circular strain gauges are situated at the front and rear of the boots, where they are affixed between two ‘floating’ metal brackets as the only point of connection between the skier and the ski. This shift in the placement of the strain gauges enabled clip-in height to be minimized, and the mass to be reoriented more distally compared to previous iterations. Each force plate could provide force and moments in three axes (medio-lateral = x, antero-posterior = y, normal = z), but only resultant forces were examined in this study.

A custom designed force collection [25] system was used in this study to provide 3-dimensional *SRF*s (see Figs 1 and 3). The system (model: ISkiSet, Sensix, Poitiers, France) comprised of two cylindrical force sensors per boot each containing six full-bridge strain gauges. Each force sensor was affixed between two brackets to create a floating frame, where the sensors were the only point of connection between the skier and the ski. The units weighed 1.2 kg each boot and raised the clip-in height by 6 mm. The units connected by cable to a small hip-mounted acquisition card (model: Jam Ingénierie, Chambery, France), which was equipped with a synchronized inertial unit (model: LSM9DS1, STMicroelectronics, Geneva, Switzerland). Raw output, acceleration, and gyroscopic movement were amplified and recorded at a sampling frequency of 200 Hz. Each set of boots underwent a lab-based calibration procedure (details explained in [31]) to provide an accurate estimate of force and torque on each axis. The result was a selection of boot-specific calibration matrices that were applied (model: MATLAB 2019B, Mathworks, MA, USA) to the raw amplitude signal from the force. The raw voltage provided by the sensors was converted into force units using the appropriate calibration matrix per boot size.

### 2.3 Data analysis

#### 2.3.1 Calibration, synchronization, filtering and turn selection processes

During the calibration procedures, athletes collected trials while balancing on each ski; the raised force plate was ‘zeroed’, with the other plate collecting athlete BM. These files were saved for later use in normalizing variables to BM, which corresponds to total system mass, including all clothing and technology equipped during the skiing trial (i.e., athlete tare BM, plus the normalized mass of the skis, force plates, boots, and personal equipment). Pre- and post- trial athletes performed a strong double limbed jump on their skis; this was to provide a clear spike on force and acceleration signals (from both the GNSS and the force-plate acquisition card) to synchronize data streams.

Force data were then combined to create a resultant value for each limb and zeroed. Data from the GNSS were resampled to match the force plates (i.e., from 10 to 200 Hz) using cubic spline interpolation, and then smoothed using a 2^nd^ order Savitzky-Golay filter with a window of 201 frames. Force-plate and GNSS data were synchronized using algorithmic cross-correlation applied around the impulses created from the athlete’s jumping motion (100 frames either side of detected peaks from the on-board accelerometers of the GNSS and force-plate acquisition card). The filtering frequency for force and positional data was first approximated based on previous research [32, 33], and then confirmed via Fast-Fourier transformation, and manual observation of the power spectral density, to remove higher frequency domain data not relevant to our analyses (e.g., vibration [34]). Subsequently, the force and positional data were filtered using 2^nd^ order low-pass Butterworth at 8 and 1.5 Hz, respectively. From this point, the synchronized and filtered data streams were separated into distinct turns using the point of inflexion in the trajectory [35]. These turn-switch events were determined by searching for the point of sign change (+ to −, or vice versa) on the triple derived trajectory data. The final temporal markers were used to cut the synchronized force and positional data into distinct matrices corresponding to each turn. 14 turns (3–16) were selected for final analysis, representing athletes having attained reasonable speed (i.e., stopped pushing with poles and skating to accelerate) to the turn directly preceding the final gate.

#### 2.3.2 Section selection

From the total analyzed turns, two main steps of analysis were chosen: i) ’*course*’ averaged variables, where the average performance over the total analyzed turns was considered as a single coherent value for testing our hypotheses, and ii) ’*sectional*’ averaged variables, where the course was separated into distinct typical sections known by ski practitioners, each characterized by the specific relief of the course and basic kinematic data (i.e., velocity and turn time): a section with the intent of gaining speed (Section 1: ‘*speed*’, turns 3–5 and 15–16), a steep section where athletes managed speed that potentially was at risk of exceeding their ‘velocity barrier’ (Section 2: ‘*overspeed*’, turns 6–10), and a flat section where athletes maintained speed (Section 3: ‘*flat*’, turns 11–14). The distinction was made separately by different experimenters possessing experience in high-level ski coaching.

#### 2.3.3 Parameter computation

For each force platform, axis data were combined into resultant force:

$$F_{\text{r}}{}_{\text{es}}\text{i}=\sqrt{F_{x}{}^{2}+F_{y}{}^{2}+F_{z}{}^{2}}$$ (1)

with *F*_res_i the magnitude of the resultant *SRF* for each force plate (outside [*F*_out_] and inside [*F*_ins_] limb per turn), and *F*_x_, *F*_y,_ and *F*_z_ the magnitude of the force components in x-, y-, and z-axis of each ski referential. The magnitude of the total force produced by both limbs onto the snow (*F*) was obtained by the sum of the magnitudes of *F*_out_ and *F*_ins_ and considered as the same magnitude as the resultant *SRF* applied to the COM. While the true magnitude of resultant *SRF* is unknown due to the impossibility of detecting the orientation of each ski in space (i.e., the true vector orientation for *F*_out_ and *F*_ins_), the error induced by any potential differences in *SRF* orientation at each ski on *F* is assumed to be minimal. The absolute difference between the limbs was calculated at each instant and expressed as a percentage of *F*:

$$diff=\left|\left(F_{\text{out}}-F_{\text{ins}}\right)\right|/F$$ (2)

**Fig 4:**
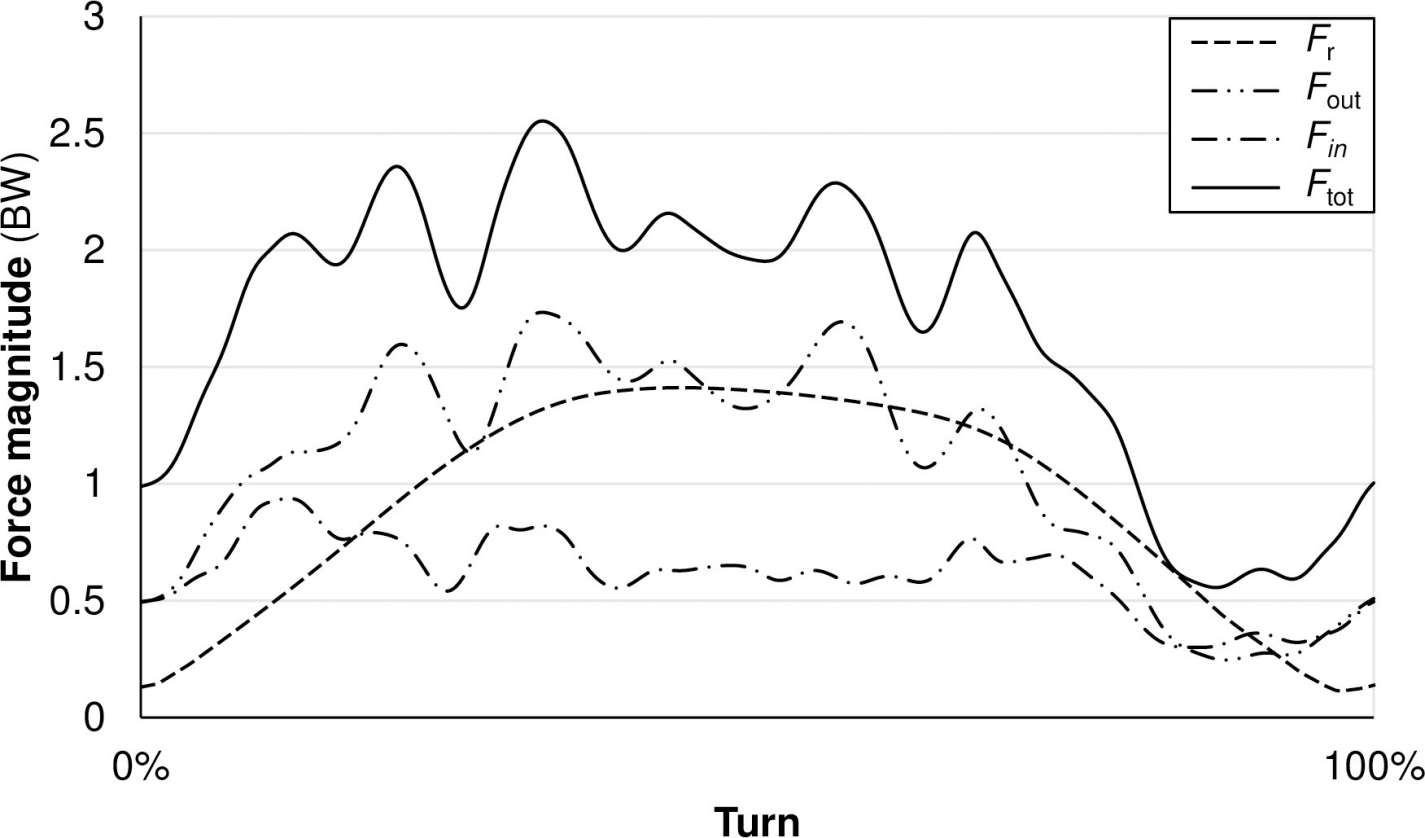
Turn-averaged force-outputs for a single high-performing athlete. Data, in this case, has been normalized as a percentage of turn duration. The solid black line represents total force output (*F*_tot_); the long-dash-dot black line represents the force output of the outside limb (*F*_in_); the long-dash-dot-dot black line represents the force output of the inside limb (*F*_out_); the short dashed black line represents radial force output (*F*_r_); all force data are displayed as a factor of BW.

From this point, the following variables were calculated for each turn: average *F* (*F*_tot_), average *diff* (*F*_diff_), peak values for *F* (*F*_max_) and for the outside and inside limbs (*F*_maxout_ and *F*_maxins_, respectively). For data provided from the GNSS unit the following turn variables were calculated: velocity at the entry (*v*_in_) and averaged (*v*_avg_), and the cumulated displacement per turn (*L*, as the trajectory based performance parameter [11], for use in the analysis model described in *Section 2*.*5*). Turn radii (*r*) was calculated by fitting three consecutive points of the trajectory (at its native frequency of 10 Hz, up sampled to 200 Hz [i.e., 60 consecutive points]) with an arc segment [36]. Radial force (*F*_r_) was computed by summing the centripetal force and the component of the bodyweight (i.e., BW in N) acting on the radial direction of the turn when angle of the slope ≠0 [37]:

$$F_{\text{r}}=\frac{v^{2}}{r}\pm g\;\text{sin}\;\alpha \;\text{cos}\;\beta$$ (3)

with α equal to slope relief, and *β* as the angle between the COM trajectory and that horizontal to the fall line. Then, *r* and *F*_r_ were averaged over each turn. The ratio of forces (*RF*) was expressed as the ratio between turn-averaged *F*_r_ and *F*_tot_. See Fig 4 for an example of the course averaged output of primary kinetic variables.

The primary macroscopic performance factor in this study was total course time (*T*). In contextualizing sectional performance, we used the change in specific mechanical energy for the turn, normalized to the velocity at entry to the turn (*v*_in_) as a velocity-based parameter [11] and as the index of performance for the turn (Δ*e*_mech_/*v*_in_ [21]):

$$\Delta e_{\text{m}}{}_{\text{ech}}/v_{\text{i}}{}_{\text{n}}=\Delta (v^{2}/2+g\cdot z)/v_{\text{i}}{}_{\text{n}}$$ (4)

where *z* corresponds to the altitude, and Δ the change between two turn-switches (i.e., the beginning and end of a turn). Δ*e*_mech_/*v*_in_ provides a valuable method of accounting for the influence of performance of previous turns [2, 9, 11], and has been used to describe the within-turn performance of a range of athletes: a higher Δ*e*_mech_/*v*_in_ (commonly, the ‘less negative’) is interpreted as less energy dissipated or more energy produced (if any), since the value is negative when energy is dissipated [2, 9].

### 2.4 Statistical analysis

Statistical analyses were performed using JASP (version: 0.11.1, JASP Team, Amsterdam, Netherlands). *T* originally violated the assumption of distribution normality (Shapiro-Wilkes score, *p =* .04), which after further inspection was due to an outlier in the dataset. While the data appeared congruent with the other scores and consistent with video observations, data from this athlete were removed due to the sensitivity of the subsequent models to outliers (final *N* = 15).

**Fig 5:**
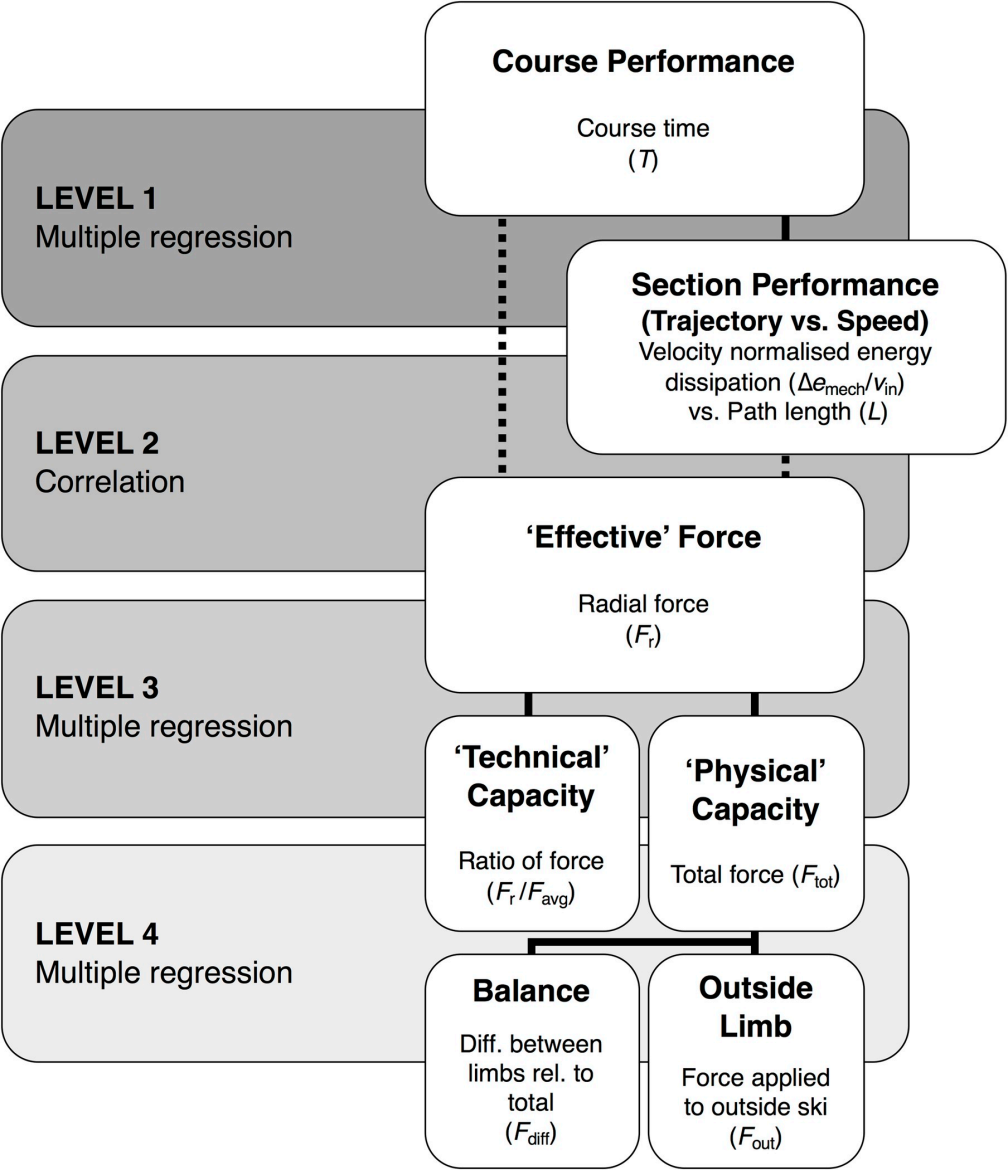
Modular outline of approach to data analysis, applied to all sectional datasets. ‘Multiple regression’ models (continuous lines) feature stepwise entry criteria, and ‘Correlation’ models (dashed lines) are Pearson’s product moment correlation coefficient.

In the first instance, key variables (turn time [*T*_turn_], *v*_in_, Δ*e*_mech_/*v*_in_, *L*, *F*_r_, *RF*, *F*_tot_, *F*_out_, *F*_ins_, *F*_diff_, *F*_max_, *F*_maxout_ and *F*_maxins_) were compared between sections using repeated measures ANOVAs with Holm post-hoc tests, and Cohen’s *d* for magnitude of difference. To characterize the determinant nature of force production, we developed a stepped analysis procedure (Fig 5) to be applied to all datasets: course, speed, overspeed, and flat. The model consisted of two primary statistical approaches: Bivariate correlation, using Pearson’s correlation (*r±*95%CIs, *p*) and stepwise multiple regression (standardized *β*, *F*, *R*^2^ change [per variable], and model *R*^2^ [adjusted], *p*) analyses. In the first level, correlational analysis tested sectional performance parameters (Δ*e*_mech_/*v*_in_ and *L*) against course performance (*T*). The following level tested the clear predictive nature of sectional *F*_r_ to sectional Δ*e*_mech_/*v*_in_ and to *T*. The next two levels of the model employed multiple regression analyses to test the relationship and contribution of force magnitude (*F*_tot_) and application effectiveness (*RF*) to *F*_r_. Subsequently, *F*_tot_ output was contextualized using the same multiple regression model to test the output and balance between the limbs (outside limb [*F*_out_] and the difference between the limbs [*F*_diff_]).

The strength of Pearson’s correlations was interpreted using threshold values of *r*<0.1, 0.3, 0.5, and 0.8 to represent trivial, small (minor), moderate, large (strong), and very large (very strong) effects, respectively [30]. For Cohen’s *d* threshold values for qualitative interpretation of effect magnitude were *d*<0.2, 0.2, 0.5, and 0.8 as trivial, small (minor), moderate, large (strong), and very large (very strong) effects, respectively. The alpha value for all tests was set at .05.

## 3 Results

For the reliability in course level kinematic data, the systematic variability was <2.1%, and the random variability was *CV*<2.86% for all variables except for Δ*e*_mech_/*v*_in_ (*CV* = 4.95%). For sectional data, systematic variability was 0.3–4.6%, and the intra-athlete variability was *CV* = 1.3–5.7% for all variables except for Δ*e*_mech_/*v*_in_, which ranged from *CV* = 6.4–9.21%. For the validity comparison with the RTK system, bias between the systems for ‘sectional’ turn groupings was -1.0–1.4%, agreement *r* = 0.69–0.97, and *CV* = 1.2–3.3% for *T*_turn_, *v*_in_, *L*, and *F*_r_ (for Δ*e*_mech_/*v*_in_, bias = -1.3%, *r* = 0.82, and *CV* = 9.1%). For full methodological details and results of both ‘sectional’ and ‘turn-by-turn’ analyses see supplementary information. The repeated-measures ANOVAs showed sectional effects in all variables except for *RF*, *F*_out_, and *F*_diff_ (see Table 1).

**Table 1:** Course and sectional descriptive data.

Variable	Sectional results (mean ± SD)								Within subject effects		Post-hoc comparisons (Cohen’s *d*)					
	Course		1. Speed		2. Overspeed		3. Flat				1 vs. 2		1 vs. 3		2 vs. 3	
	mean	±SD	mean	±SD	mean	±SD	mean	±SD	*F*	*p*	*d*	*p*	*d*	*p*	*d*	*p*
*T*_turn_ (s)	1.60	±0.10	1.70	±0.13	1.64	±0.094	1.44	±0.094	112	< .001	0.887	.004	3.196	< .001	3.719	< .001
*v*_in_ (m/s)	15.60	±0.89	15.23	±1.07	15.88	±0.98	16.31	±0.96	15	< .001	-1.009	.003	-1.429	< .001	-0.465	.093
*L* (m)	26.56	±0.34	27.53	±0.43	27.14	±0.78	24.63	±0.69	79	< .001	0.473	.089	2.979	< .001	2.279	< .001
*Δe*_mech_/*v*_in_ (Js/kg/m)	-5.59	±0.52	-5.45	±0.73	-6.49	±0.63	-4.65	±0.50	64	< .001	1.797	< .001	-1.092	< .001	-3.262	< .001
*Δalt* (m)	9.33	±0.44	9.54	±0.53	10.25	±0.68	7.91	±0.75	62	< .001	-0.824	.007	1.690	< .001	3.596	< .001
*F*_tot_ (BW)	1.60	±0.16	1.55	±0.18	1.62	±0.16	1.65	±0.16	22	< .001	-1.095	.002	-1.687	< .001	-0.500	.073
*F*_out_ (BW)	1.06	±0.12	1.04	±0.14	1.07	±0.12	1.08	±0.12	2	.169	-0.366	.357	-0.454	.302	-0.171	.518
*F*_ins_ (BW)	0.54	±0.10	0.50	±0.10	0.56	±0.11	0.58	±0.11	9	< .001	-0.993	.004	-1.053	.003	-0.223	.402
*F*_diff_	0.32	±0.084	0.35	±0.10	0.31	±0.09	0.30	±0.09	2	.113	0.559	.145	0.511	.145	0.094	.721
*F*_max_ (BW)	3.48	±0.38	3.21	±0.37	3.80	±0.42	3.44	±0.43	37	< .001	-2.300	< .001	-0.589	.039	1.243	< .001
*F*_maxout_ (BW)	2.49	±0.30	2.33	±0.29	2.648	±0.32	2.505	±0.34	23	< .001	-1.740	< .001	-0.979	.004	0.766	.010
*F*_maxins_ (BW)	1.42	±0.30	1.289	±0.24	1.545	±0.39	1.438	±0.36	8	.002	-0.939	.008	-0.629	.057	0.449	.104
*F*_r_ (BW)	1.06	±0.11	1.00	±0.11	1.07	±0.11	1.10	±0.16	6	.008	-1.091	.003	-0.804	.015	-0.216	.216
*RF*	0.68	±0.056	0.66	±0.051	0.67	±0.063	0.68	±0.093	<1	.531	-0.261	.990	-0.258	.990	-0.128	.990

Descriptive data are displayed as mean ± standard deviation; within subject effects are described by the *F* test (*F)* and *p-value* (*p*); posthoc comparisons are represented in Cohen’s *d* with Holm correction; Course, data averaged over total analysis window (14 turns); 1. Speed, turn averaged data from speed section; 2. Overspeed, turn averaged data from overspeed section; 3. Flat, turn averaged data from flat section; *T*_turn_, average turn time per section; *v*_in_, velocity measured at entry to turn; *L*, cumulated path length; Δ*e*_mech_/*v*_in_, change in specific mechanical energy, relative to entry velocity; *F*_r_, radial force; *F*_tot_, averaged total force output; *F*_out_, averaged force output for outside limb; *F*_ins_, averaged force output for inside limb; *F*_diff_, difference in force between outside and inside limb; *F*_max_, averaged maximum total force output; *F*_maxout_, averaged maximum force output for outside limb; *F*_maxins_, averaged maximum force output for in limb; *RF*, ratio of radial force to averaged force; s, seconds; m/s, meters per second; m, meters; Js/kg/m, joule seconds per kilogram per meter; BW, bodyweight.

### 3.1 Level 1 –relationships between course and sectional performance parameters

Δ*e*_mech_/*v*_in_ over the course, and in speed and overspeed sections, was negatively predictive of *T* (Table 2). *L* did not significantly improve the model in any case. In the flat section, no combination of variable models clearly predicted *T*.

**Table 2:** Course performance (*T*) explained by turn modifiable factors averaged for each section (analysis ‘Level 1’).

Predictor	*stand*. *β*	*p*	*R*^*2*^ *change*	*F*	*df*	*model R*^2^
Course				32	1, 13	0.686
Δ*e*_mech_/*v*_in_	-0.842	< .001	0.708			
*L*	-	-	-			
1. Speed				37	1,13	0.717
Δ*e*_mech_/*v*_in_	-0.859	< .001	0.737			
*L*	-	-				
2. Overspeed				13	1,13	0.460
Δ*e*_mech_/*v*_in_	-0.706	.003	0.498			
*L*	-	-				
3. Flat				-	-	-
Δ*e*_mech_/*v*_in_	-	-				
*L*						

*stand*. *β*, beta-weight in standardized units; *p*, *p-value*; *F*, *F* test; *df*, degrees of freedom; Grey background denotes no clear model prediction (*p*>.05) of model to course performance; Course, data averaged over total analysis window (14 turns); 1. Speed, turn averaged data from speed section; 2. Overspeed, turn averaged data from overspeed section; 3. Flat, turn averaged data from flat section; Δ*e*_mech_/*v*_in_, average change in specific mechanical energy, per entry velocity of each turn; *L*, averaged cumulated displacement per turn; bold denotes first variable to enter into predictive model.

### 3.2 Level 2 –relationships between course and sectional performance parameters and radial force

*F*_r_ produced throughout the course and in speed and overspeed sections was negatively correlated with *T*, and positively correlated with section specific Δ*e*_mech_/*v*_in_ (see Table 3). In the case of the flat section, *F*_r_ was related to *T*, but not with Δ*e*_mech_/*v*_in_.

**Table 3:** Pearson’s product moment correlation coefficients between radial force (*F*_r_) and sectional (Δ*e*_mech_/*v*_in_) and course performance (*T*) (analysis ‘Level 2’).

Sectional models	*Pearson’s r*		*P*	95% CI	
				Lower	Upper
Relationship between *T* and sectional *F*_r_					
*Course*	-0.826	*very strong*	< .001	-0.940	-0.543
*1*. *Speed*	-0.831	*very strong*	< .001	-0.942	-0.554
*2*. *Overspeed*	-0.772	*strong*	< .001	-0.920	-0.430
*3*. *Flat*	-0.582	*strong*	.023	-0.843	-0.100
Relationship between sectional Δ*e*_mech_/*v*_in_ and sectional *F*_r_					
*Course*	0.720	*strong*	.002	0.329	0.900
*1*. *Speed*	0.647	*strong*	.009	0.202	0.871
*2*. *Overspeed*	0.554	*strong*	.032	0.058	0.831
*3*. *Flat*	0.261		.348	-0.290	0.682

Grey background denotes no clear prediction of independent variable; Course, data averaged over total analysis window (14 turns); CI, confidence interval; 1. Speed, turn averaged data from speed section; 2. Overspeed, turn averaged data from overspeed section; 3. Flat, turn averaged data from flat section; *T*, time in seconds between start and finish of course; *F*_r_, radial force; Δ*e*_mech_/*v*_in_, change in specific mechanical energy normalized to entry velocity of each turn; Note: all variables with the exception of *T* are averaged per the relevant section.

### 3.3 Level 3 –contribution of ‘technical’ and ‘physical’ capacities

In all sections both *F*_tot_ and *RF* were predictive of *F*_r_ (both variables clearly contributed to the predictive model) and explained the quasi-entire variance (*R*²>0.99) in *F*_r_ (see Table 4). Over the course and in the three sections, both *RF* and *F*_tot_ explained an important part of variance in *F*_r_ (*R*² change from 0.36 to 0.64) and presented high normalized effects (*β*>0.63), although the order of the two predictors in the explained variance and prediction strength changed with each section.

**Table 4:** Multiple regression analysis results assessing the importance of *F*_tot_ and *RF* to predict *F*_r_, specific to the course and each section (analysis ‘Level 3’).

Sectional model	*stand*. *β*	*R*^*2*^ *change*	*F*	*df*	*model R*^2^
Course			449	1, 12	0.986
***F***_**tot**_	**0.939**	0.497			
*RF*	0.739	0.491			
1. Speed			649	1,12	0.989
***F***_**tot**_	**1.003**	0.635			
*RF*	0.631	0.356			
2. Overspeed			736	1,12	0.991
***F***_**tot**_	**0.921**	0.412			
*RF*	0.811	0.580			
3. Flat			382	1,12	0.982
***RF***	**0.918**	0.600			
*F*_tot_	0.637	0.385			

*stand*. *β*, beta-weight in standardized units; *p*, *p-value*; *F*, *F* test; *df*, degrees of freedom; Course, data averaged over total analysis window (14 turns); 1. Speed, turn averaged data from speed section; 2. Overspeed, turn averaged data from overspeed section; 3. Flat, turn averaged data from flat section; *F*_r_, radial force; *F*_tot_, averaged resultant ground reaction force; *RF*, ratio of radial force to averaged force (from force plate); bold denotes first variable to enter into predictive model; all *p* < .001.

### 3.4 Level 4 –contribution of force to the outside limb and the balance between limbs to total force output

In all sections *F*_tot_ was predicted by a model which (by order) positively correlated to *F*_out_, and negatively correlated to *F*_diff_ (see Table 5). In contextualizing these results, additional Pearson’s correlations unveiled positive correlations between the force output for both limbs individually and *F*_tot_ in sections (*r* = 0.613–0.835, *p* < .001–.015), and no clear relationship in any section between *F*_ins_ and *F*_out_ (*r* = -0.002–0.13, *p* = .645–.996).

**Table 5:** Multiple regression analysis results assessing the importance of *F*_out_ and *diff* to explain *F*_tot_ specific to the course and each section (analysis ‘Level 4’).

Sectional Model	*stand*. *β*	*R*^*2*^ *change*	*F*	*df*	*model R*^2^
Course			1013	1,12	0.995
***F***_**out**_	**1.079**	0.642			
*F*_diff_	-0.657	0.354			
1. Speed			1607	1,12	0.996
***F***_**out**_	**1.179**	0.697			
*F*_diff_	-0.647	0.299			
2. Overspeed			563	1,12	0.988
***F***_**out**_	**1.044**	0.551			
*F*_diff_	-0.728	0.438			
3. Flat			1824	1,12	0.996
***F***_**out**_	**1.179**	0.517			
*F*_diff_	-0.924	0.479			

*stand*. *β*, beta-weight in standardized units; *F*, *F* test; *df*, degrees of freedom; Course, data averaged over total analysis window (14 turns); 1. Speed, turns with moderate relief with the target of gaining speed; 2. Overspeed, turns with high relief with the target of managing built up speed; 3. Flat, turns with low relief and perceived low difficulty; *F*_out_, averaged resultant ground reaction forces applied to outside limb in the turns; *F*_diff_, turn-averaged ratio of absolute difference between force applied to inside and outside limb and the total force; bold denotes first variable to enter into predictive model; all *p* < .001.

## 4 Discussion

This study showed that GS course performance in experienced to World Cup level skiers is better explained by an ability to minimize the dissipation of energy per section, rather than the distance traveled. This same finding was generally applicable within distinct sections of the course, except for a flat section in which neither normalized speed energy dissipation nor trajectory parameters could clearly distinguish athletes. Radial force output was strongly or very strongly related to course performance, and strongly related to less energy dissipation in most sections. Elevated production of radial force was explained firstly by a greater capacity to produce total force, and secondly to apply a greater proportion of this force in the radial direction. Greater force production capability was linked to an increased capability to generate force to the outside ski, and subsequently to apply force to the inside ski (i.e., a reduced difference between the limbs). In general, each of our hypotheses were confirmed, but were contextualized with stronger, weaker, or even absent relationships in specific sections; notably, the course performance was predicted by neither trajectory nor speed performance parameters in the flat section, and performance within this section (energy dissipation) was not clearly predicted by radial force production.

In general, our findings support other analyses [11] that preference methods of attaining and maintaining velocity while minimizing energy loss (e.g., carving [1–3, 9]), rather than aiming to reduce path length [2, 8]. In our results course, speed, and overspeed sectional Δ*e*_mech_/*v*_in_ explained a substantial proportion of variance in *T* (*R*^2^ = 0.46–0.67, *p <* .003), whereas *L* did not. The overall lack of clear association between *L* and performance (course, speed, and overspeed sections) points to better athletes selecting strategies that enabled them to minimize dissipation of energy regardless of the trajectory. This could be a product of higher performing athletes possessing enhanced perceptual, technical, and physical capabilities, resulting in enhanced pattern flexibility to select the optimal trajectory per turn and entry characteristics. The lack of clear predictive value of speed and trajectory parameters in the flat section might highlight that these variables do not represent the best measure of performance in this section or that different strategies in this section can be associated to similar performances. Notably, the much lower relief of this flat section and potential energy production behaviors of the athletes could feasibly reduce the predictive ability of Δ*e*_mech_/*v*_in_ [38]. Instead, performance might be more clearly predicted by ‘determinant’ parts of the course [9, 16] that stress technical and physical athletic limits. The minorly weakened relationship between sectional Δ*e*_mech_/*v*_in_ and *T* in the overspeed section might be a result of athlete tactics; notably the purposeful dissipation of energy to benefit later turns [2]. Such a tactic would create a negative result in the turn/section in which the dissipation occurred (in our analyses, weaken the negative correlation) but produce a net positive on overall performance. The first turn of the overspeed section follows a delayed gate, which might have necessitated some dissipation of energy [14] to avoid mistakes and elevate performance in later turns. Lastly, it is worth considering that Δ*e*_mech_/*v*_in_ is sensitive to computational and methodological errors, albeit at acceptable levels in our analyses (i.e., *CV*<10%). Consequently, the error associated with this variable might have prevented the detection of otherwise significant interesting effects.

In general, there were strong to very strong associations between lower course times and greater *F*_r_. Although there is little empirical data on *F*_r_ in skiing, our findings are congruent with evidence that performance is largely determined by attaining a high average velocity [5], and carving represents the optimal approach to skiing at speed while minimizing ski-snow friction [24]. Logically it should follow that athletes exhibiting greater *F*_r_ would likewise minimize energy dissipation (i.e., a less negative Δ*e*_mech_/*v*_in_), however this was not the case in all sections. While a clear and substantial relationship existed between elevated *F*_r_ and Δ*e*_mech_/*v*_in_ over the course and in speed and overspeed sections, this relationship was unclear in the flat section; which might suggest greater variability in the technique adopted to generate *F*_r_ (e.g., energy gain). Likewise, the moderate association observed between *F*_r_ and Δ*e*_mech_/*v*_in_ in the steep overspeed section, might suggest tactfully shedding some speed and circumspectly maintaining or even increasing *F*_r_ in later turns (observed in conserved strong relationship between *F*_r_ and *T* in overspeed section). In any case, athletes who developed high sectional *F*_r_ were likely to attain faster course times, and thus logically would present a higher velocity barrier.

In all sections, the combination of total *SRF* magnitude (*F*_tot_) and the capability to apply a larger proportion of that output in the radial direction (*RF*) significantly predicted *F*_r_. On average, 68% of *F*_tot_ was applied radially, with much greater values observed in overspeed and flat compared to the speed section. In this case, *RF* represents a ratio of ‘mechanical effectiveness’ in which better athletes theoretically apply a greater proportion of their total output in an efficient manner (i.e., higher proportion of *F*_tot_ radially). A similar model has been presented in sprint running and cycling, where it has been subsequently used to better understand the determinants of acceleration performance and orient training goals [39]. Our results show that the weighted contribution of *F*_tot_ and *RF* to *F*_r_ changes in explanatory magnitude and hierarchical order depending on the section. On the one hand, in the speed section *F*_tot_ had much greater explanatory power than *RF* (*R*^2^ = 0.64 and 0.36, respectively). One possibility is that better athletes generated force in response to targeted dissipation, and transient and random vibrations [7] that might otherwise disrupt the motion of lesser capable skiers to maintain optimal skiing technique (and generate high levels of *F*_r_). In the flat section *F*_r_ was much more strongly associated with *RF* than *F*_tot_ (*R*^2^ = 0.60 vs. 0.39, respectively), which might highlight the reduced necessity for targeted dissipation with athletes further from their velocity ceilings; the substantially lower Δ*e*_mech_/*v*_in_ in this section strengthens this theory, and it is possible that athletes performed positive muscular work and gained additional energy [38], which served to both partly compensate energy dissipation and to elevate *F*_r_. Several studies have recorded positive values of energy loss in skiing, suggesting that some energy was regained [9, 14, 19] during rhythmic turns. However, in the overspeed section and over the total course, *F*_tot_ and *RF* were quite balanced in their contributions to elevated *F*_r_ (e.g., course, *R*^2^ = 0.50 and 0.49, respectively). These findings have interesting implications for practice, since in general better athletes both technically orient a larger proportion of force to perform at preferable trajectories and speeds (i.e., elevate carving, as a product of improved *F*_r_) and display generally enhanced force output on the skis. However, since the absolute physical aptitude of the skiers were not measured in this study (rather, the ability to exhibit output within the constraints of skiing) it is unclear whether force output on the skis (radial, or otherwise) represents or is limited by i) maximum athletic capacity, ii) maximum athletic capacity mediated by various constraints (technique, tactic etc.), or iii) a combination of these and other factors. Evidence of near maximal muscular activation during skiing [40] and enhanced physical capacity for force in skiers [6] would support the theory of physical capability being a determinant factor, but further evidence is needed.

Athletes produced force at approximately a 2:1 ratio between the outside and inside limbs. While greater force applied to the outside limb was hierarchically the strongest predictor of global force output in all sections (*p* < .001), a strong inverse relationship with the *difference* between limbs was observed (*p* < .001) in all sections; specifically, a globally higher total force output was explained by a higher force output of the operative limb and an associated more equal application of force between the limbs (i.e., disproportionately greater contribution of the inside limb). These findings were further corroborated with individual correlations between force produced to both the inside and outside limbs (*F*_ins_ and *F*_out_, respectively) and sectional *F*_tot_, without relationship *between* the limbs. There are several possible explanations for this finding, each of which assume a relationship between total force and carving [24] (tested in *Level 3*). There are two scenarios representing better performance (i.e., a higher *F*_r_): tighter turns (i.e., lower radius and shorter trajectory) and turns performed at higher speeds. In the former example, a skier aiming to reduce their turn radius must first increase the edging angle of the ski [41]. In this aim, athletes incline their body laterally towards the center of the turn, where applying force at this increased edging angle acts to bend and carve the skis through the snow resulting in a tighter radius [24]. The capability to turn with tighter radii depends on the capability to produce forces to the mechanically advantageous outside limb at a biomechanically unique position. Increasing edging angle requires inclination of the upper body towards the turn center where some application of force to the inside ski is needed to maintain control and active balance [42]. However, evidence showing supra-maximal (>maximum voluntary isometric contraction) levels of activation [43] indicates turns that require very high levels of *F*_r_ may supersede the single-limb force-production capabilities of an athlete; in such a circumstance, there is no other option than to apply a proportion of force using the other limb (or to disperse energy). In any case, this finding highlights the necessity of conditioned force output for the inside limb, which is notable given the disparate knee and hip angles at which these forces are produced between the two limbs [23, 44]. In physical conditioning, high-level athletes might focus on the development of force application at angles of the knee/hip complex relevant to the outside ski and the inside ski (~-20° for the inside limb, using a non-carving approach [43] and -34° in recent literature [23]). Such an approach should elevate the force production of the lower limbs in total (i.e., increase *F*_tot_) and allow for more flexibility in performing turns that require both greater force application in general and an enhanced contribution of the inside limb (i.e., more balanced force profile).

### 4.1 Limitations

Athletes were required to wear a variety of relatively unfamiliar equipment which may have affected their performance. While steps were taken to resolve this factor (e.g., athletes provided with their preference of boots, familiarized and confirmed the setup did not unreasonably compromise their capabilities) athletes may not have performed at their peak. The methodological approach of averaging multiple turns was chosen to reduce error [30], provide a more macroscopic view of performance, and to highlight the capability of detecting worthwhile differences in performance using more accessible GNSS technology. While our results support the conclusions of more detailed analyses [11], including those from other disciplines [2, 9, 14], turn-by-turn analyses afforded by higher accuracy positional devices (e.g., low-cost RTK) would aid in further contextualizing the relationship between force output and performance in skiing. While in this study carving is considered the ‘optimal’ technique, there are instances in which athlete deviate from this approach and preference other strategies. These instances might result in a more beneficial overall performance for the athlete [5, 16], but are not directly considered in our model.

## 5 Conclusions

Our results support other analyses that better GS performances are predicted by lower energy dissipation at higher speeds. Higher radial forces are related to performance and are determined by both total force production and an enhanced ability to orient a greater proportion radially. The contribution between production and orientation of force varies depending on course characteristics. Athletes who apply more force (and potentially perform better) adopt techniques which elevate total force output via the outside limb, and subsequently a proportionally greater contribution of the inside limb. These results strengthen evidence for enhanced force-output capacities in ski racers and might provoke conditioning focus for the inside limb. While both ‘technical’ and ‘physical’ force qualities appear determinant in GS skiing, we cannot conclude whether the athletes were truly exhibiting their physical limits; further detailed analyses, including cross-examination of off-snow tests of physical output, should help clarify these points. Increased force-output capacity, in combination with adopting techniques to improve force application effectiveness (*RF*) on the skis, should raise radial force capacity and allow athletes greater physical and technical freedom in trajectory selection. Lastly, these results may be context, technology, or athlete specific, and should be tested in greater depth.

## Supporting information

S1 Material Pilot-data of sports GNSS validity using a low-cost Real-Time Kinematic (RTK) system as a criterion. (DOCX)

S1 Fig Supporting information Fig 1. Low-cost Real-Time Kinematic (RTK) device. Clockwise from top: battery pack, RTK compatible receiver, padded bag to house electronics around waist, portable computer system, and high-fidelity antenna.
(TIF)

S2 Fig Supporting information Fig 2. Systematic and random error between the RTK device and the digitized ground-track data in longitude data. Figure depicts data collected from four trials of the RTK device, relative to that from the digitized ground plan of the mountain coaster, displayed here as longitude normalized to latitude. The top figure displays the longitude of the four runs (black lines) and the ground track (dashed line) as a function of the displacement in latitude. The bottom graph displays the mean systematic variability between the RTK and ground track in m (black), and the variability (light grey SD lines) along the latitude. Note: the variability is extremely low, so the SD bars cross the mean error.
(TIF)

S3 Fig Supporting information Fig 3. Systematic and random error between the RTK device and the digitized ground-track data in altitude data. Figure depicts data collected from four trials of the RTK device, relative to that from the digitized ground plan of the mountain coaster, displayed here as altitude normalized to latitude. The top figure displays the altitude of the four runs (black lines) and the ground track (dashed line) as a function of the displacement in latitude. The bottom graph displays the mean systematic variability between the RTK and ground track in m (black), and the variability (light grey SD lines) along the latitude.
(TIF)

S4 Fig Supporting information Fig 4. ’Exploded’ view of RTK and ground-track data. Data here corresponds to an ‘exploded’ section of RTK and ground-track data displayed in Figs 4 and 5 (top and bottom, respectively). The purpose here is to show that the graphic does indeed display four separate trajectories (black lines) alongside the digitized ground path of the coaster (dashed line).
(TIF)

S5 Fig Supporting information Fig 5. Bland-Altman plot of variables calculated from the RTK and GNSS devices. vertical pairs of plots correspond to each variable, with the horizontal corresponding to either turn-by-turn analysis (a), or ‘sectional’ analyses (b). y-axes display the difference between the two devices for the corresponding variable, with the grey lines representing limits of agreement (±1.96 SD). *v*_avg_, averaged velocity; *v*_in_, velocity at turn entry; *L*, cumulated distance travelled; *F*_r_, radial force; Δ*e*_mech_/*v*_in_, change in specific mechanical energy normalized to velocity at turn entry; s, seconds; m/s, meters per second; m, meters; N/kg, Newtons per kilogram; Js/kg/m, joule seconds per kilogram per meter. Note: turn-time is not displayed in this figure, since the data were not easily readable (majority of differences were identical, at either 0 or 0.1 s).
(TIF)
