# Supplementary material for: Force output in giant-slalom skiing: A practical model of force application effectiveness
Source: PLoS One. 2021 Jan 14;16(1):e0244698. doi: 10.1371/journal.pone.0244698 (PMC7808649; doi:10.1371/journal.pone.0244698)
Supplement: S1 Material — (DOCX) [file pone.0244698.s001.docx]

Pilot-data of sports GNSS validity using a low-cost Real-Time Kinematic (RTK) system as a criterion

# Introduction

In alpine skiing, measurements of trajectory are valuable for biomechanics research and for practitioners to provide worthwhile feedback to athletes. Unfortunately, the nature of alpine skiing challenges collecting such data. Several accurate capture methods exist (e.g. motion capture), but their complexity often results in brief capture windows (1-3 turns). Global Navigation Satellite Systems (GNSS) have been proposed as a feasible means of providing a variety of important metrics for performance, ranging from simple time and velocity data, to more sensitive parameters like change in mechanical energy (1). As such, the ongoing development of increasingly accessible GNSS technology is of interest (2). Regardless, since substantial variability exists among and between GNSS units (3), it is important to ensure systems provide accurate and reliable enough data to detect worthwhile differences in performance metrics.

The aim of this pilot-study was to test the validity of a sports GNSS device to measure various performance parameters in alpine skiing. To this end, we performed two experiments: 1) a validity and reliability study for our custom compiled ‘low-cost’ Real-Time Kinematic system (RTK) on a known and repeatable trajectory against ground plot information, and 2) a criterion validity comparison between the RTK system and a sports GNSS system for individual turns, and multiple turns collapsed into ‘sectional’ averages.

# Methods and materials

## Procedures

### Mountain coaster experiment

An RTK unit was fixed on a mountain coaster rail system, that circulated throughout an overcast morning of experimentation. The rail system was at a French ski resort (Saint François Longchamp, France; altitude ~1700 m), and consisted of a ~996 m track that turned back and forth alongside a ski slope, along which a two-seated sledge circulated at a velocity controlled by the riders (via a braking system). The sledge path was practically immovable, comprising of a central metal rail and two adjoining parallel rails.

The RTK antenna was placed in the center of a padded seat at the rear of the sledge above the riders’ heads, and the electronics in a small bag affixed to the bottom of the seat. A base station was attached to the roof of a vehicle positioned in an unobstructed location near the bottom of the course. We performed 4 separate trials in the morning over three hours in overcast metrological conditions. Each run had different velocity traces (and time) due to non-repeatable braking patterns. The mountain coaster was selected for this test for three interrelated reasons: 1) it provided a quasi-identical trajectory across multiple runs, which significantly improved the ease with which numerous trials can be compared for reliability, 2) it shares key similarities to skiing, including a mountainous environment, similar speeds, and quasi-similar alternating turning styles, and 3) while unable to access a higher quality reference system against which to validate the system, we were provided with a digital high-resolution ground-track document that allowed digitization for overlay and comparison.

### Skiing experiment

Athletes performed two trials of a marked giant-slalom course while equipped with a custom-built RTK and a sports GNSS system. The experimental collection was performed during the final stages of habitual training, and as such the athletes were already familiar with the course, warmed-up, and ready for maximum effort skiing. Athletes performed 1-2 race-paced runs, after which various performance metrics were derived from individual turns and sections (consecutive grouped turns) and then compared to test the validity of the sports-GNSS device. Athletes provided written informed consent, the operational procedures were approved by the ethics board of University Savoie Mont Blanc, and procedures were in congruence with the Declaration of Helsinki.

The athletes were female regional-level skiers (*N*=4; age, 19±1 yr). Testing occurred on hard groomed snow, and during stable environmental conditions (~1 hr, during the morning). Brush gates were set by a federation coach to model a giant slalom setup (20 gates) on a moderate grade consistent grade slope (~17º relief). Athletes wore their own boots and used their own ski gear and equipment (e.g. helmet, goggles, and back protectors). Athletes were equipped with two distinct systems that provided positional data: 1) a sports GNSS system (model: Stadium tracker, Mac-Lloyd Sport, Paris, France), and 2) a custom-built RTK (as the ‘criterion’ measure).

Two separate ‘rover’ RTKs were worn by athletes during the testing period in a small backpack, with the antenna attached to the helmet in the same manner as the sports GNSS. An identical unit was used as a base/reference station that was fixed to a tripod on a flat open area above the testing site and remained stationary and unobstructed throughout the testing procedures.

## Equipment

For both experiments, position was provided by RTK (see Supplementary Figure 1) compatible GNSS unit/s built from the following componentry: a high-fidelity antenna (model: ANN-MB, uBlox, Thalwil, Switzerland; gain, 28±3.0 dB) and RTK compatible receiver (model: M8T, uBlox, Thalwil, Switzerland), wired to a small portable computer (model: RasberryPi zero, Kubii, Lyon, France) and small battery pack (~100 g). The units were capable of reading signals from a variety of satellite systems (American [GPS], Russian [GLONASS], European [Galileo], and Chinese [BeiDou]), on the L1 frequency at 10 Hz. Each unit cost ~150 euros, which was 5-10× less costly than comparable commercial alternatives.

For the skiing experiment, an additional sports GNSS system provided positional data from the Russian (GLONASS) and American (GPS) satellite systems at a down-sampled rate of 10 Hz fused with data from an onboard inertial measurement unit (4). The units were lightweight (~20 g per unit) and were worn attached to the helmet of the user using commercial Velcro.

**----**

**Supporting information 2. Figure 1**. Low-cost Real-Time Kinematic (RTK) device. Clockwise from top: battery pack, RTK compatible receiver, padded bag to house electronics around waist, portable computer system, and high-fidelity antenna.

----

## Data analysis

For each experiment, rather than providing ‘real-time’ feedback, the data from the RTK rovers and base-station were extracted and analyzed post-operatively using programs from the RTKLIB (version: 2.4.3 b33) directory (5). The distinct navigational data from the rover and base station were combined to provide kinematic positioning solutions, with an elevation mask of 20° to exclude low-strength satellites (per the manufacturer’s instructions). Where used, the sports GNSS data were downloaded using the manufacturer supplied software. From this point, the results were imported and processed using MATLAB (model: R2019b, Mathworks Inc., Natick, Massachusetts, USA).

### Mountain coaster

The downhill portion of the run was separated from the total course for analysis, just after the release of the carriage for the first run (i.e., just after the first low speed turn). The rationale for this decision was that the beginning, uphill and straight portions of the start of the course were covered with roofing which impaired signal acquisition, in addition to comprising mostly slow or stationary periods of motion. Just before the downhill portion the RTK system transitioned to a fixed solution. Data were synchronized at a distinct geographical point and zeroed in all axis. The raw, unfiltered data were used for comparisons.

A reference ground track was generated from the information obtained from a high-quality digital document. Specifically, distinct data regarding the measurement scale, and the center axis of the sledge track was extracted using image digitization in MATLAB. Due to the lack of exact geographic positioning, the resulting longitude-latitude data for the ground track was rotated to fit the overlaid trajectories of the RTK system using cross-correlation. Note: the plans from which the ground track was digitized were preliminary building plans which did not necessarily represent the exact dimensions of the finished structure, and represented the center of the rails upon which the sledge ran (i.e., the RTK antenna was ~1.5 m above the rails, and the rails ranged from <1 m from the ground to >3 m). As such, this comparison features several limitations which we will discuss in the interpretations of the results.

### Skiing

Positional data (longitude, latitude, and altitude) were smoothed (11 frame Savitzky-Golay), and then low-pass filtered (1 Hz 2^nd^ order Butterworth). The files were initially cut at just before and after the trial using velocity-time data. From this stage, individual turns were extracted using the point of inflexion in the triple derived longitude positional data (6) as distinct turn-switch events (i.e. the end of one turn, and the beginning of the proceeding turn). Turns during which the RTK system did not provide the most accurate outcome solution (i.e., ‘float’ rather than ‘fixed’ = four turns in total) were excluded from the analysis for both units. The final data selected for analysis consisted of 16-17 turns per athlete (total of 93 turns), depending on the ‘tactical’ approach to the first and last turn cycles (e.g., early skidding on the last turn was excluded).

### Parameter computation

#### Mountain coaster

For the RTK validity test, only longitude, latitude, and altitude were extracted for comparison across different runs. No other variables were extracted, mostly due to different operator speeds across runs voiding the possibility of assessing other interesting variable sets (e.g., energy behaviors). The difference in longitude and altitude data from the ‘criterion’ digitized ground track in m were computed along position in latitude for each of the four trials.

#### Skiing experiment

Per each distinct data stream, for each turn the following variables were computed: turn-entry and averaged velocity (*v*_in_ and *v*_avg_, respectively), cumulated displacement (*L,* see (7)), average radial force (8), and the difference in specific mechanical energy normalized to the velocity at turn entry (∆*e*_mech_⁄*v*_in_ (9)).

Two distinct data analysis approaches were employed to test the validity of the devices: 1) Data from all possible turns were individually compared between the units (e.g. *L* turn 1 for each athlete, GNSS vs RTK), and 2) data from each athlete (16 turns, with first turn excluded where necessary) were grouped into 4 consecutive turns, averaged and then compared in the same manner (e.g. *L* averaged across turns 1-4 for each athlete, GNSS vs RTK).

## Statistical analysis

For the mountain coaster, we adopted a simplified approach to detecting systematic and random error and variability to measure validity and reliability, respectively. Specifically, along each dimension of latitude, the mean and standard deviation of the four units for altitude and longitude were calculated. Subsequently the mean, and standard deviation of the mean represented the systematic error, and variability across the entire run, respectively. To represent the random error and variability of the RTK units, the mean and standard deviation of the standard deviation were similarly calculated across the entire run.

Descriptive data are presented as means±standard deviation. The RTK and GNSS data were compared using linear regression, Pearson’s correlations, and typical error. Overall bias between the methods was assessed using the mean difference between the practical (GNSS) and that determined from a calibration equation via regression analysis of the criterion (RTK) and reported in raw units ±95% confidence intervals. Strength of agreement between the two measures was assessed using Pearson’s correlation coefficient (*r*) on the log transformed data, and threshold values of 0.3, 0.5, 0.7, and 0.9 to represent weak, moderate, strong, and very strong agreement, respectively. Typical error of estimate as the coefficient of variation (*CV*%) provided a measure of error magnitude. Analysis was repeated on all turns in the first instance, and then on the grouped turns from all athletes.

# Results

For the mountain coaster, the mean systematic error compared to the ground track in longitude data was 0.697 m, with the variability along the course in this error being 0.616 m. For altitude, the mean systematic error compared to the ground track was 0.431 m, with the variability along the course in this error being 0.366 m. For longitude data, the mean random error was 23.18 mm, with the variability along the course of this error being 7.47 mm. For altitude, the mean random error was 185.42 mm, with the variability along the course of this error being 66.05 mm. The longitude- and altitude-latitude data are presented in Figure 2 and 3, respectively.

----

Supporting information 3. Figure 2. Systematic and random error between the RTK and the digitized ground-track in longitude

Figure depicts data collected from four trials of the RTK, relative to that from the digitized ground plan of the mountain coaster, displayed here as longitude normalized to latitude. The top figure displays the longitude of the four runs (black lines) and the ground track (dashed line) as a function of the displacement in latitude. The bottom graph displays the mean systematic variability between the RTK and ground track in m (black), and the variability (light grey SD lines) along the latitude. Note: the variability is extremely low, so the SD bars cross the mean error.

----

Supporting information 4. Figure 3. Systematic and random error between the RTK and the digitized ground-track in altitude

Figure depicts data collected from four trials of the RTK, relative to that from the digitized ground plan of the mountain coaster, displayed here as altitude normalized to latitude. The top figure displays the altitude of the four runs (black lines) and the ground track (dashed line) as a function of the displacement in latitude. The bottom graph displays the mean systematic variability between the RTK and ground track in m (black), and the variability (light grey SD lines) along the latitude.

----

**Supporting information 5. Figure 4.** 'Exploded' view of RTK and ground-track data

Data here corresponds to the ‘exploded’ section of RTK and ground-track data displayed in Figure 4 and Figure 5 (top and bottom, respectively). The purpose here is to show that the graphic does indeed display four separate trajectories (black lines) alongside the digitized ground path of the coaster (dashed line).

----

Full descriptive data and results for the skiing test are presented in Supplementary Table 1, with plots of the bias ±95% limits of agreement (associated to Bland Altman plots) for the two levels of analyses displayed in Supplementary Figure 5. Turn-by-turn data were typically low bias (<1.5% for all variables, except *∆e*_mech_/*v*_in_ [4.5%]), with *moderate* to *very strong* agreement in all variables. In general, variability was low (*CV*<5%) except for *F*_r_ and *∆e*_mech_/*v*_in_ which exhibited *large* and *very large* error (7.0 and 12.9%), respectively.

----

Supporting information 6. Figure 5. Bland-Altman plot of the differences in variables calculated from the RTK and GNSS. vertical pairs of plots correspond to each variable, with the horizontal corresponding to either turn-by-turn analysis (a), or ‘sectional’ analyses (b). y-axes display the difference between the two devices for the corresponding variable, with the grey lines representing limits of agreement (±1.96 SD). *v*_avg_, averaged velocity; *v*_in_, velocity at turn entry; *L*, cumulated distance travelled; *F*_r_, radial force; ∆*e*_mech_/*v*_in_, change in specific mechanical energy normalized to velocity at turn entry; s, seconds; m/s, meters per second; m, meters; N/kg, Newtons per kilogram; Js/kg/m, joule seconds per kilogram per meter. Note: turn-time is not displayed in this figure, since the data were not easily readable (majority of differences were identical, at either 0 or 0.1s).

----

Supplementary Table I. Comparison of variables calculated from positional data collected from RTK and GNSS units

| Turn variables | RTK | | GNSS | | RTK vs GNSS | | |
| --- | --- | --- | --- | --- | --- | --- | --- |
|  | Mean | ±SD | Mean | ±SD | *Overall raw bias* (*95% CI*) | *Pearson’s* ( *r* ) | *Typical error estimate* ( % ) |
| Turn-by-turn (*N*=93) | | | | | | | |
| *T* (s) | 1.58 | ±0.13 | 1.58 | ±0.15 | 0.0022 (-0.012; 0.017) | 0.86 | 4.2 |
| *v*_avg_ (m/s) | 17.93 | ±1.24 | 17.96 | ±1.40 | 0.036 (-0.069; 0.14) | 0.91 | 3.1 |
| *v*_in_ (m/s) | 18.06 | ±1.54 | 17.89 | ±1.63 | -0.17 (-0.31; -0.034) | 0.93 | 3.6 |
| *L* (m) | 29.92 | ±1.69 | 29.86 | ±2.13 | -0.054 (-0.24; 0.17) | 0.62 | 4.5 |
| *F*_r_ (N/kg) | 8.19 | ±0.73 | 8.34 | ±1.03 | 0.15 (0.0081; 0.30) | 0.69 | 7.0 |
| *∆e*_mech_*/v*_in_ (Js/kg/m) | -3.72 | ±0.52 | -3.66 | ±1.02 | 0.051 (-0.23; 0.13) | 0.51 | 12.9 |
| Grouped turns (*N*=23) | | | | | | | |
| *T* (s) | 1.58 | ±0.08 | 1.58 | ±0.08 | 0.0022 (-0.0058; 0.010) | 0.97 | 1.2 |
| *v*_avg_ (m/s) | 18.17 | ±0.43 | 18.19 | ±0.65 | 0.018 (-0.16; 0.20) | 0.78 | 1.5 |
| *v*_in_ (m/s) | 18.35 | ±0.51 | 18.17 | ±0.73 | -0.18 (-0.35; -0.000029) | 0.84 | 1.5 |
| *L* (m) | 30.45 | ±1.77 | 30.39 | ±1.99 | -0.066 (-0.34; 0.21) | 0.95 | 1.8 |
| *F*_r_ (N/kg) | 8.23 | ±0.36 | 8.35 | ±0.60 | 0.13 (-0.064; 0.32) | 0.69 | 3.3 |
| *∆e*_mech_*/v*_in_ (Js/kg/m) | -3.83 | ±0.65 | -3.81 | ±0.80 | 0.022 (-0.19; 0.15) | 0.82 | 9.1 |
| RTK*,* Global Navigation Satellite System with Real-Time Kinematic post-processing capabilities; GNSS, Global Navigation Satellite System; *SD*, standard deviation; *CI*, confidence intervals; *v*_avg_, averaged velocity; *v*_in_, velocity at turn entry; *L*, cumulated distance travelled; *F*_r_, radial force; *∆e*_mech_*/v*_in_, change in specific mechanical energy normalized to velocity at turn entry; s, seconds; m/s, meters per second; m, meters; N/kg, Newtons per kilogram; Js/kg/m, joule seconds per kilogram per meter. | | | | | | | |

Sectional data understandably shared similar bias scores to turn-by-turn analyses (<1.4%), but agreement was substantially improved with all but *F*_r_ representing *strong* or *very strong* agreement (*moderate* agreement for *F*_r_). Similarly, error was markedly reduced by adopting a sectional approach, with all variables *CV*<9.1%.

# Discussion and conclusion

Despite systematic error in our measurements, we can infer validity and excellent reliability in our RTK. The sports GNSS unit provided sufficient estimates of several key turn-averaged performance parameters in ski racing. While surprisingly the values provided from turn-by-turn analyses appear generally acceptable, adopting a sectional approach markedly reduced systematic error. As such, practitioners and researchers might benefit from adopting a sectional approach in their analyses and athlete feedback; particularly when examining variables such as *∆e*_mech_*/v*_in_ and *F*_r_ that are valuable in contextualizing skiing performance but suffer from inflated computational errors. Such an approach could feasibly allow the use of common sports GNSS technology into alpine skiing practice and research; albeit requiring post-processing and considering remaining error rates.

The results from our mountain coaster experiment depict low levels of random error and variability in both z- and x-axis data (altitude, and longitude). In fact, we assume these error rates are inflated, as the rails of the coaster slightly flexed as a function of the force applied to the track. Moreover, while the z-axis error was understandably higher (3), the necessity of normalizing along y-axis (latitude) data for our comparison is a clear explanation for overly inflated variability. In any case, these results are encouraging for the integration of similar technologies into alpine skiing practice, given the similar characteristics under which this experiment was performed (e.g., alpine environment, similar speeds and trajectory characteristics).

While at face value the mountain coaster validity data appear average, there are several explanations for these results. Namely, the position of the RTK antenna was above that inferred from the ground track of the coaster, and along the track the rails were set to pivot the coaster back and forth. As such, this pivoting created an offset in the longitude-latitude position between the top of the sledge (i.e., the antenna position) and the ground track. This pivoting typically occurred during turning (i.e., to offset *F*_r_), and probably explains the characteristic tracking of the RTK trajectory inside each turn (see the clear and repeated offsets in Figure 2). Moreover, since the digitized plan was not final, presumably there are some minor differences between our plot and that finalized on the course. For these reasons, we believe we can infer validity from our results, however validation using a true criterion device is needed. Particularly, concerns still exist regarding the capability of such units to detect appropriate targeting solutions in various environments, since a known issue with such ‘low-cost’ units is their disposition to fail to find a fixed solution, or detect a ‘false’ fix (3, 10). More research using similar updated technology—notably integrating higher quality antennae and higher sampling frequencies in congruence with the concerns of previous investigations (10)—are warranted.

Among the limitations of this pilot approach were both units being mounted to the head, which was chosen for practicality (i.e., difficulty attaching both units in a small space, without compromising athlete safety), and for the likelihood of clear satellite coverage for both units. Moreover, our RTK system has not been validated due to the lack of access to technology with higher resolution. While tests of RTK systems (high (11) and low-cost (12)) report excellent accuracy, future applied research is needed in alpine skiing.

Sufficiently accurate performance data can be measured from sports GNSS units, particularly when paired with a ‘sectional’ approach to analyzing and presenting data. While acceptable, error associated with force and energy behaviors was still present and should be considered when interpreting study results. Our results suggest that while moderate quality GNSS units have their limitations and should be interpreted in tandem to the inflated error compared to more accurate tools and measurements, they might still represent a means of providing valuable performance metrics in research and practice.

# References

1. Supej M. Differential specific mechanical energy as a quality parameter in racing alpine skiing. J Appl Biomech. 2008;24(2):121-9.

2. Supej M, Spörri J, Holmberg HC. Methodological and practical considerations associated with assessment of alpine skiing performance using global navigation satellite systems. Front Sports Act Living. 2020;1.

3. Gilgien M, Spörri J, Limpach P, Geiger A, Müller E. The effect of different Global Navigation Satellite System methods on positioning accuracy in elite alpine skiing. Sensors. 2014;14(10):18433-53.

4. Gloersen O, Kocbach J, Gilgien M. Tracking performance in endurance racing sports: Evaluation of the accuracy offered by three commercial gnss receivers aimed at the sports market. Front Physiol. 2018;9:1425.

5. Takasu T, Yasuda A. Development of the low-cost RTK-GPS receiver with an open source program package RTKLIB 2009. Available from: <http://gpspp.sakura.ne.jp/paper2005/isgps_2009_rtklib_revA.pdf>.

6. Adelsberger R, Aufdenblatten S, Gilgien M, Tröster G. On bending characteristics of skis in use. Procedia Engineering. 2014;72:362-7.

7. Spörri J, Kröll J, Schwameder H, Müller E. The role of path length- and speed-related factors for the enhancement of section performance in alpine giant slalom. Eur J Sport Sci. 2018:1-9.

8. Jentschura UD, Fahrbach F. Physics of skiing: The ideal carving equation and its applications. Can J Phys. 2004;82(4):249-61.

9. Supej M, Holmberg HC. A new time measurement method using a high-end global navigation satellite system to analyze alpine skiing. Res Q Exerc Sport. 2011;82(3):400-11.

10. Gussiås HS. Prestasjonsanalyse med GNSS i alpint (Performance analysis with GNSS in alpine skiing). Norway: Norwegian University of Life Sciences; 2017.

11. Supej M. 3D measurements of alpine skiing with an inertial sensor motion capture suit and GNSS RTK system. J Sports Sci. 2010;28(7):759-69.

12. Valente DSM, Momin A, Grift T, Hansen A. Accuracy and precision evaluation of two low-cost RTK global navigation satellite systems. Comput Electron Agric. 2020;168.
