# Supplementary figures and images for: Force output in giant-slalom skiing: A practical model of force application effectiveness
Source: PLoS One. 2021 Jan 14;16(1):e0244698. doi: 10.1371/journal.pone.0244698 (PMC7808649; doi:10.1371/journal.pone.0244698)

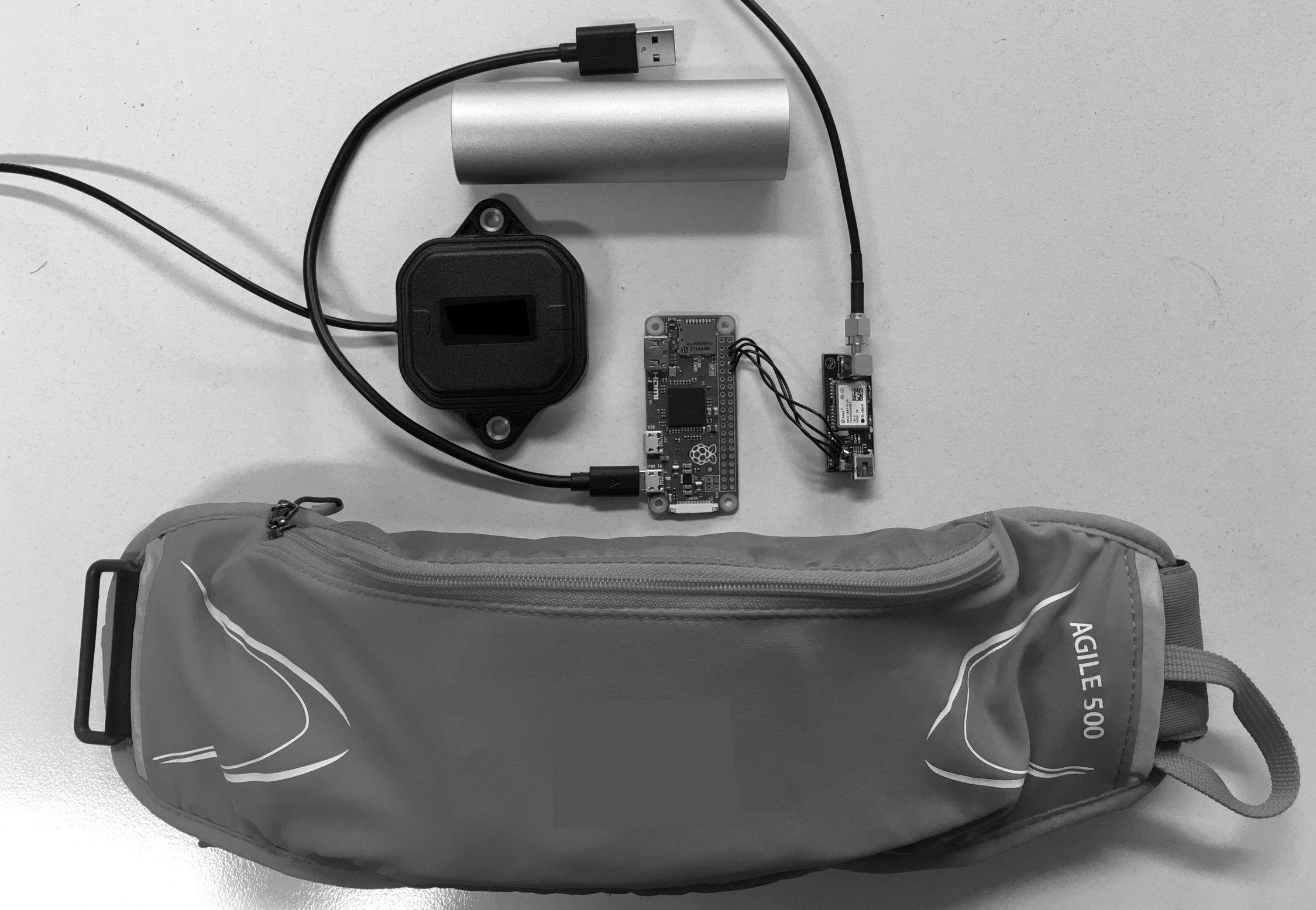

Supplement: S1 Fig — Low-cost Real-Time Kinematic (RTK) device. Clockwise from top: battery pack, RTK compatible receiver, padded bag to house electronics around waist, portable computer system, and high-fidelity antenna. (TIF) [file pone.0244698.s002.tif]

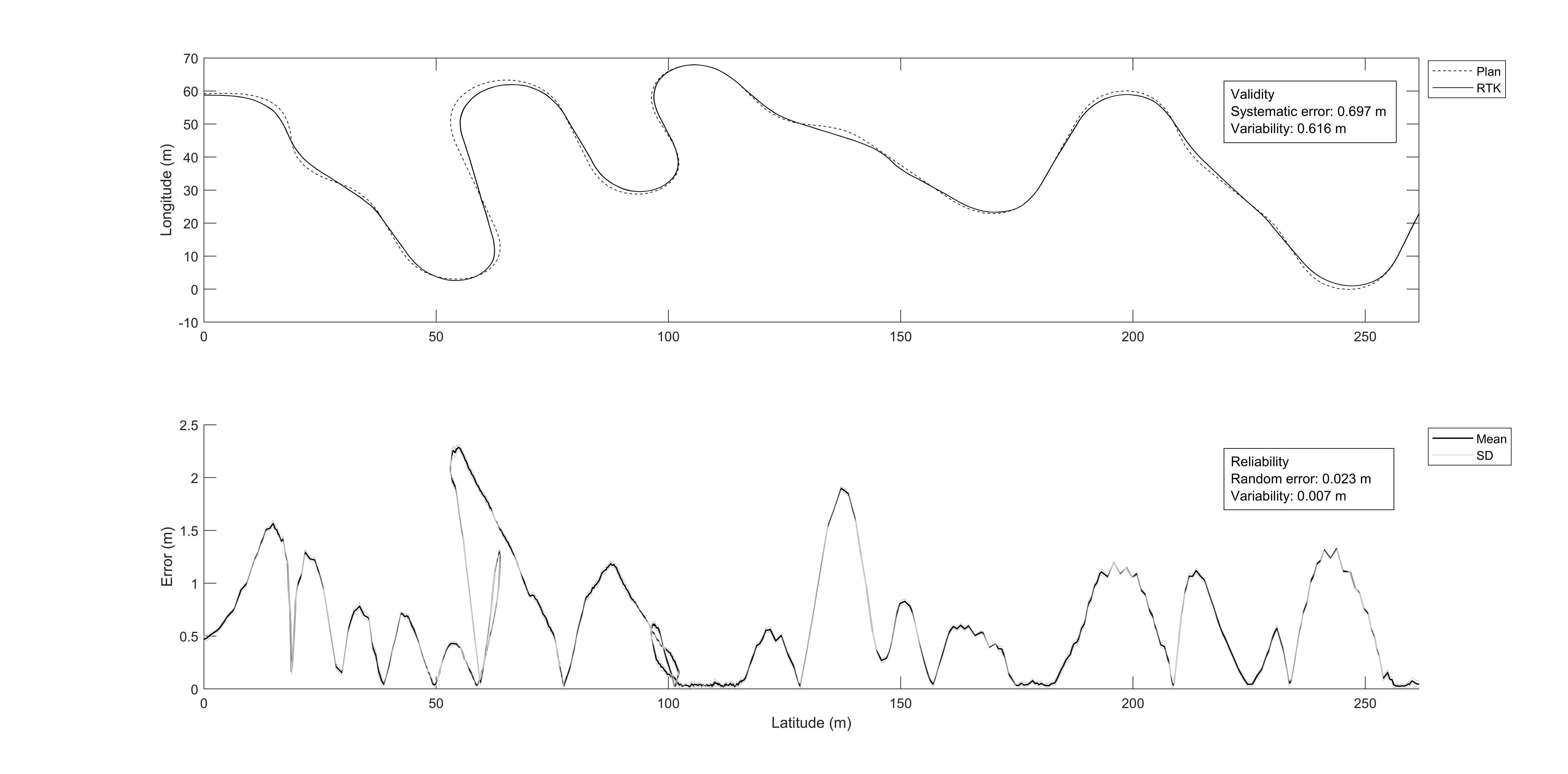

Supplement: S2 Fig — Systematic and random error between the RTK device and the digitized ground-track data in longitude data. Figure depicts data collected from four trials of the RTK device, relative to that from the digitized ground plan of the mountain coaster, displayed here as longitude normalized to latitude. The top figure displays the longitude of the four runs (black lines) and the ground track (dashed line) as a function of the displacement in latitude. The bottom graph displays the mean systematic variability between the RTK and ground track in m (black), and the variability (light grey SD lines) along the latitude. Note: the variability is extremely low, so the SD bars cross the mean error. (TIF) [file pone.0244698.s003.tif]

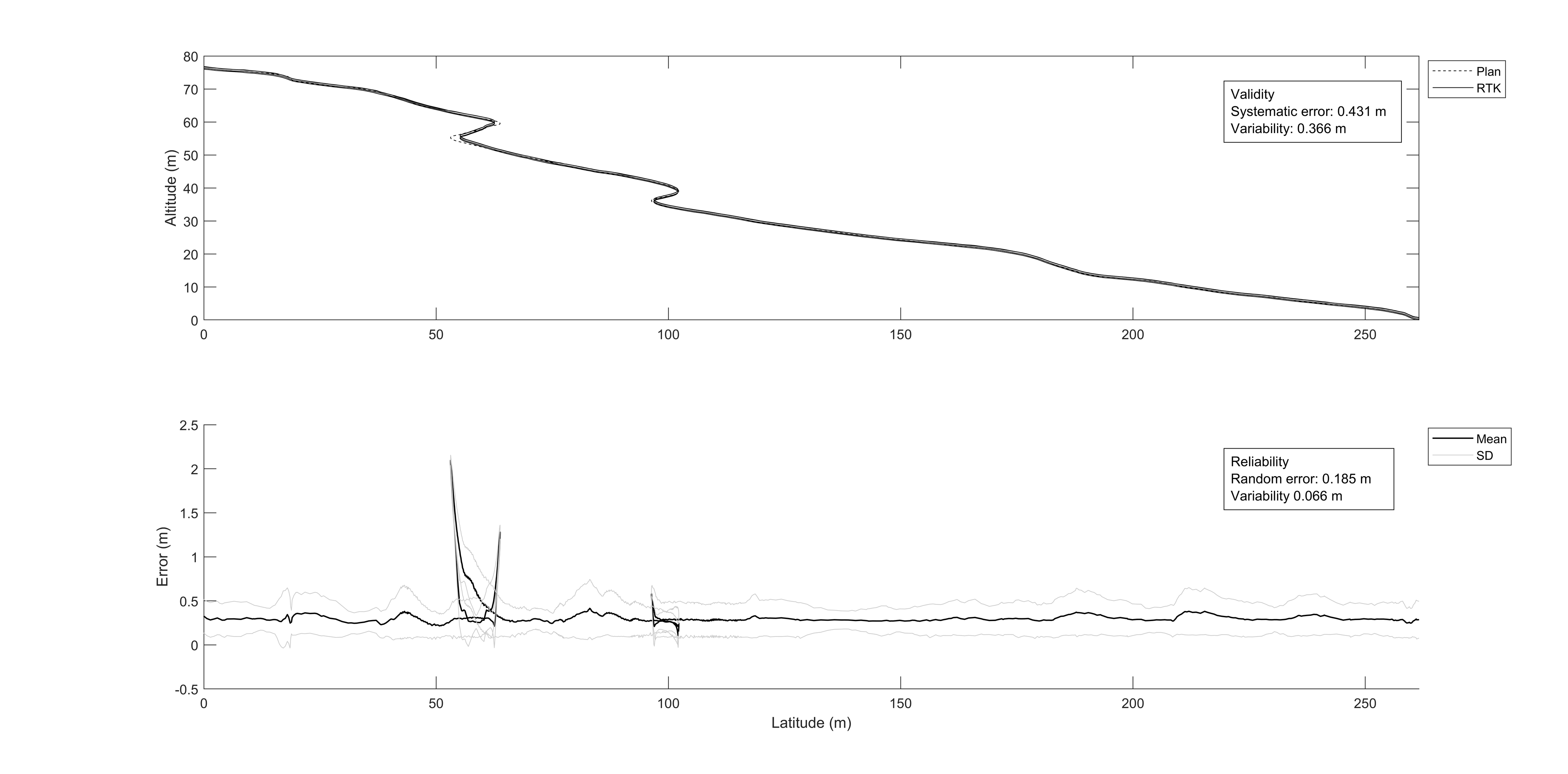

Supplement: S3 Fig — Systematic and random error between the RTK device and the digitized ground-track data in altitude data. Figure depicts data collected from four trials of the RTK device, relative to that from the digitized ground plan of the mountain coaster, displayed here as altitude normalized to latitude. The top figure displays the altitude of the four runs (black lines) and the ground track (dashed line) as a function of the displacement in latitude. The bottom graph displays the mean systematic variability between the RTK and ground track in m (black), and the variability (light grey SD lines) along the latitude. (TIF) [file pone.0244698.s004.tif]

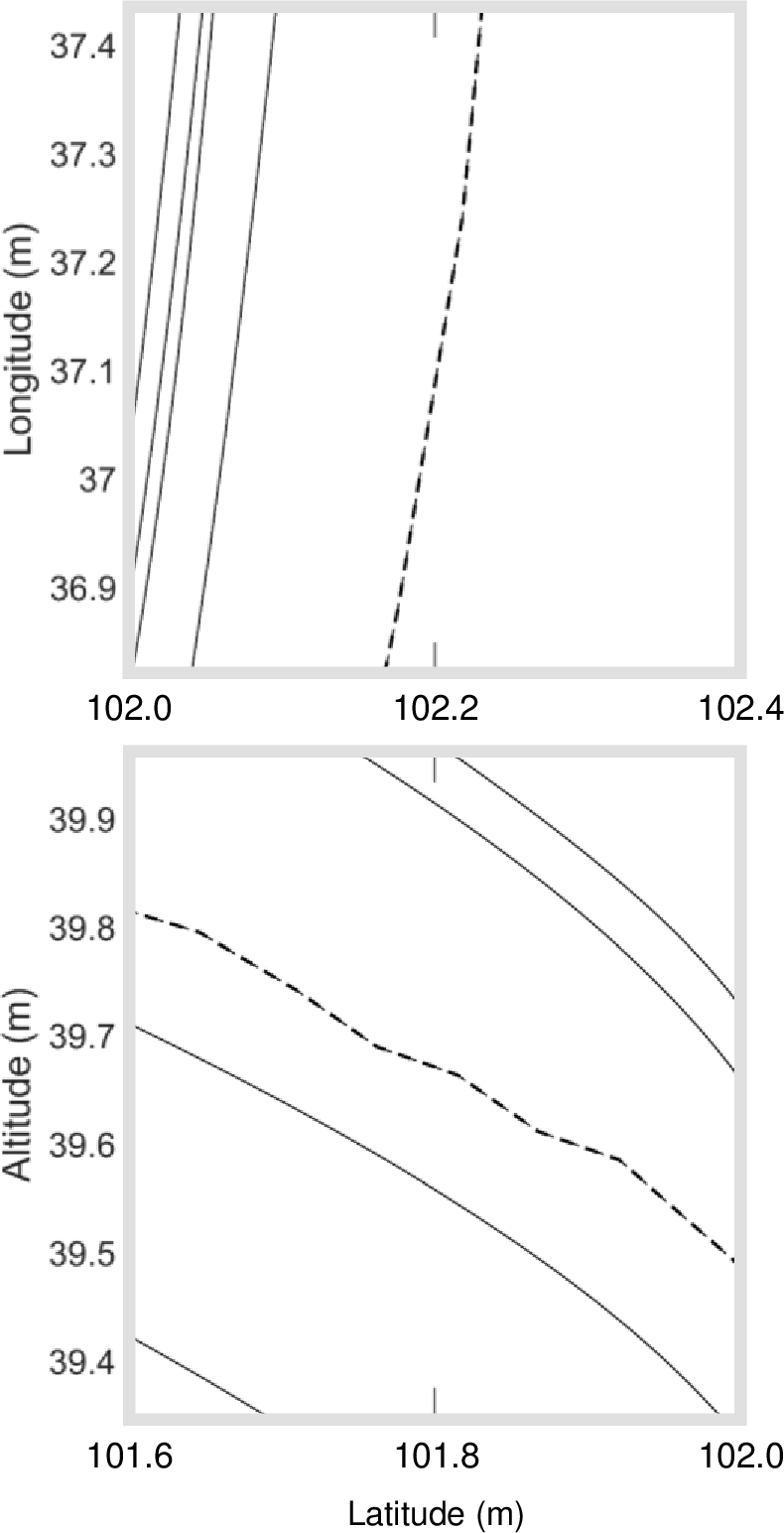

Supplement: S4 Fig — ’Exploded’ view of RTK and ground-track data. Data here corresponds to an ‘exploded’ section of RTK and ground-track data displayed in Figs 4 and 5 (top and bottom, respectively). The purpose here is to show that the graphic does indeed display four separate trajectories (black lines) alongside the digitized ground path of the coaster (dashed line). (TIF) [file pone.0244698.s005.tif]

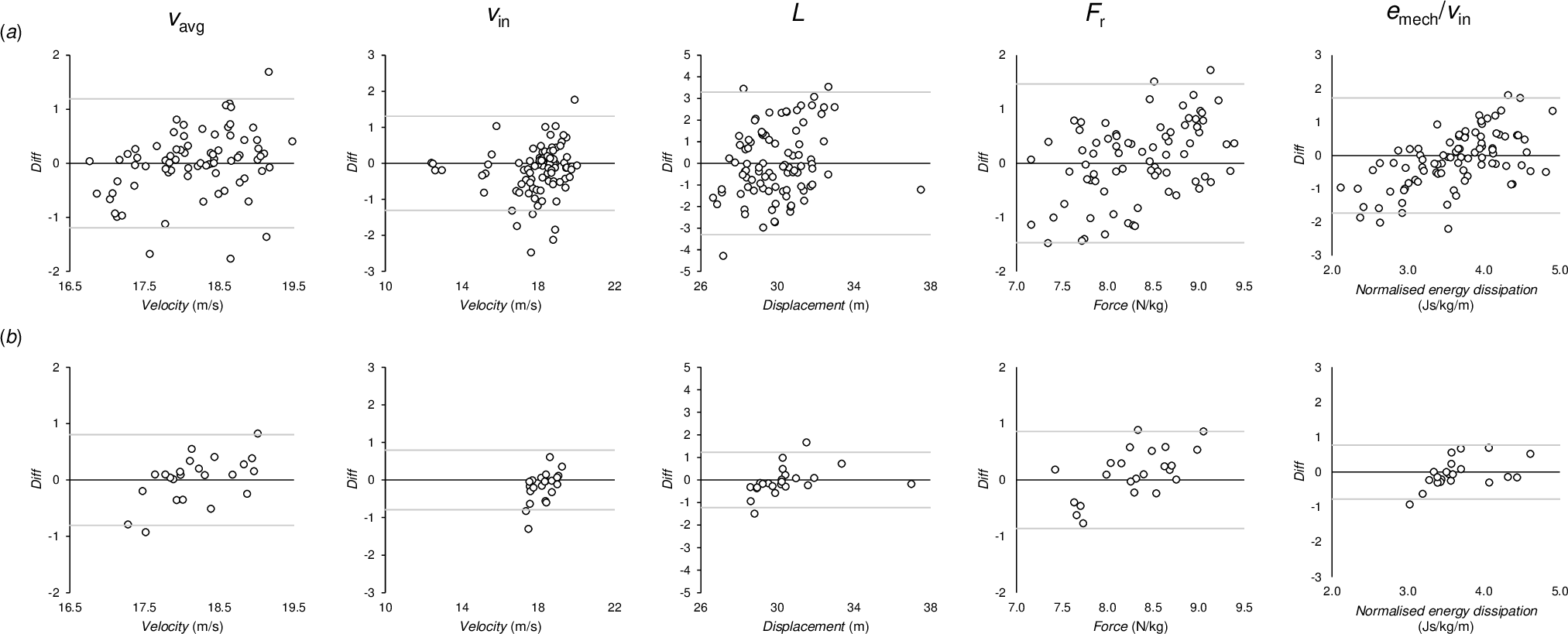

Supplement: S5 Fig — Bland-Altman plot of variables calculated from the RTK and GNSS devices. vertical pairs of plots correspond to each variable, with the horizontal corresponding to either turn-by-turn analysis (a), or ‘sectional’ analyses (b). y-axes display the difference between the two devices for the corresponding variable, with the grey lines representing limits of agreement (±1.96 SD). vavg, averaged velocity; vin, velocity at turn entry; L, cumulated distance travelled; Fr, radial force; Δemech/vin, change in specific mechanical energy normalized to velocity at turn entry; s, seconds; m/s, meters per second; m, meters; N/kg, Newtons per kilogram; Js/kg/m, joule seconds per kilogram per meter. Note: turn-time is not displayed in this figure, since the data were not easily readable (majority of differences were identical, at either 0 or 0.1 s). (TIF) [file pone.0244698.s006.tif]
